# Adaptive drift and barrier-avoidance by a fly-forage migrant along a climate-driven flyway

**DOI:** 10.1186/s40462-021-00272-8

**Published:** 2021-07-13

**Authors:** Wouter M.G. Vansteelant, Laura Gangoso, Willem Bouten, Duarte S. Viana, Jordi Figuerola

**Affiliations:** 1 Estación Biológica de Doñana, CSIC. Cartuja TA-10, Edificio I, Calle Américo Vespucio, s/n, 41092 Sevilla, Spain; 2 Institute for Biodiversity and Ecosystem Dynamics (IBED), University of Amsterdam, Sciencepark 904, 1098 XH Amsterdam, The Netherlands; 3 Department of Biodiversity, Ecology and Evolution. Faculty of Biology, Complutense University of Madrid, C/ José Antonio Novais 2, 28040 Madrid, Spain; 4 German Center for Integrative Biodiversity Research (iDiv), Deutscher Platz 5e, Halle-Jena-Leipzig, Leipzig, Germany

**Keywords:** Aeroecology, Biometeorology, Bird migration, Flight behaviour, Orientation, Weather

## Abstract

**Background:**

Route choice and travel performance of fly-forage migrants are partly driven by large-scale habitat availability, but it remains unclear to what extent wind support through large-scale wind regimes moulds their migratory behaviour. We aimed to determine to what extent a trans-equatorial fly-forage migrant engages in adaptive drift through distinct wind regimes and biomes across Africa. The Inter-tropical Front (ITF) marks a strong and seasonally shifting climatic boundary at the thermal equator, and we assessed whether migratory detours were associated with this climatic feature. Furthermore, we sought to disentangle the influence of wind and biome on daily, regional and seasonal travel performance.

**Methods:**

We GPS-tracked 19 adult Eleonora’s falcons *Falco eleonorae* from the westernmost population on the Canary Islands across 39 autumn and 36 spring migrations to and from Madagascar. Tracks were annotated with wind data to assess the falcons’ orientation behaviour and the wind support they achieved in each season and distinct biomes. We further tested whether falcon routes across the Sahel were correlated with the ITF position, and how realized wind support and biome affect daily travel times, distances and speeds.

**Results:**

Changes in orientation behaviour across Africa’s biomes were associated with changes in prevailing wind fields. Falcons realized higher wind support along their detours than was available along the shortest possible route by drifting through adverse autumn wind fields, but compromised wind support while detouring through supportive spring wind fields. Movements across the Sahel-Sudan zone were strongly associated to the ITF position in autumn, but were more individually variable in spring. Realized wind support was an important driver of daily travel speeds and distances, in conjunction with regional wind-independent variation in daily travel time budgets.

**Conclusions:**

Although daily travel time budgets of falcons vary independently from wind, their daily travel performance is strongly affected by orientation-dependent wind support. Falcons thereby tend to drift to minimize or avoid headwinds through opposing wind fields and over ecological barriers, while compensating through weak or supportive wind fields and over hospitable biomes. The ITF may offer a climatic leading line to fly-forage migrants in terms of both flight and foraging conditions.

**Supplementary Information:**

The online version contains supplementary material available at 10.1186/s40462-021-00272-8.

## Introduction

Migrating birds must negotiate highly variable as well as dynamic atmospheric conditions during their global wanderings. In many species, the routes of a single individual may lie hundreds if not thousands km’s apart in consecutive years, despite migrant birds tend to return to the same location each year. Such route flexibility is likely adaptive, enabling birds to cope with annual variation in local weather conditions and resource availability [1–3]. Nevertheless, climatic conditions and prevailing winds change in a somewhat seasonally predictable manner across the globe. For example, while westerlies dominate the mid-latitudes, easterly trade winds prevail in the Hadley cells and converge at the Inter-tropical Convergence Zone (ITCZ) along the seasonally shifting thermal equator [4]. Because wind has a strong impact on flight costs [5] such prevailing winds and other persistent circulation patterns can create reliable freeways as well as persistent blockades for aerial migrants at regional to continental scales [6–10]. Studies integrating biologging data with atmospheric models generally reveal some alignment of seasonal loop migrations with prevailing winds across marine [11–13] as well as terrestrial environments [14–17]. For landbirds this is especially true over ecological barriers - where exhaustion from battling adverse winds can have lethal consequences [18–20]. Furthermore, it is expected that birds tolerate more drift in the early stages of their migration, and increasingly (over) compensate for the incurred drift as they approach their destination [6, 7]. However, birds can theoretically engage in adaptive drift anywhere along the flyway, minimizing transport costs by (partially) drifting in strong and opposing winds, and (over) compensating for previously incurred (or ‘anticipated’) drift in areas with weak or following winds [7, 15, 21].

In the context of trans-African migration, the Inter-Tropical Front (ITF) marks a particularly sharp boundary between seasonal climates along the thermal equator, which moves northward during the spring migration, and southward during the autumn migration [22–24]. This boundary is often also called the ITCZ. However, the ITF which occurs over continental Africa is structurally distinct from the ITCZ which occurs over the ocean [22, 23]. The ITF marks the separation between areas under the influence of strong and dry north-easterly desert winds (the so-called Harmattan) and weaker and humid monsoon flows coming from the Atlantic and Indian Ocean, and like the ITCZ, the ITF is characterized by weak horizontal winds. However, while the main band of convective precipitation coincides with the ITCZ over the ocean, the northern limit of the monsoon rain belt lags some 100–250 km south of the ITF during its northward advance in spring [24]. These monsoon dynamics are key drivers of vegetation growth and the emergence of insect prey for migrant birds in Africa’s savannas, and various authors have pointed to the significance of the seasonally shifting ITF for trans-African migrants [25, 26]. Yet to our knowledge no studies have empirically tested how the ITF affects route choice of migrant birds in Africa, and still few migration studies have explicitly assessed trans-African migration routes in the context of large-scale seasonal wind regimes (but see [10] and references therein).

In this study, we assess how prevailing winds around the thermal equator and across distinct biomes mould trans-equatorial migration routes and performance patterns of Eleonora’s Falcons *Falco eleonorae*, a fly-forage migrant that exhibits great flexibility in route choice and performance across Africa. The picture emerging from over a decade of tracking studies across the Mediterranean breeding range of Eleonora’s falcons is that of an increasingly pronounced zig-zag autumn migration pattern towards more western populations. Adults thereby depart in a south- to southeast direction towards Madagascar from their respective colonies, turning to the southwest over the Sahara, and reorienting to Madagascar after reaching vegetated areas in the Sahel [27–31]. In spring, adults from all colonies tend to return along a more eastern, and in most cases shorter route, with most individuals using stop-over sites to the south and east of the Ethiopian Highlands, in the Horn of Africa. This seasonal loop migration pattern occurs in part because Eleonora’s falcons minimize the distance flown over sea via the Mozambique Channel in opposing autumn winds, while making ocean-crossings in excess of 1000 km from northern Madagascar to East Africa in supportive spring winds [29–32]. However, route choice has rarely been studied in the context of the wind support that is available across the entire flyway [31], and studies that tested the effect of experienced wind conditions on daily and seasonal performance have yielded contradictory results [29, 33]. This may be due to the confounding influence of ecological barrier effects and foraging opportunities across different biomes. Indeed, biomes are likely to be characterized by distinct climatic conditions and wind regimes. However, the relative importance of landscape and wind in shaping falcon routes and performance -and of fly-forage migrants more generally- remains unclear.

Our study population is situated at the western limit of the species’ range, so that falcons must cross the entire breadth of Africa to and from their Malagasy wintering grounds [34]. This makes Canarian Eleonora’s falcons an ideal model to study the response of a trans-equatorial fly-forage migrant to the shifting position of the ITF, and determine to what extent adaptive drift through prevailing winds shapes their seasonal route choice and performance across multiple biomes. Based on the adaptive drift framework, we expect Eleonora’s falcons to deviate from the shortest possible route (i.e., the great-circle route GCR) by (over) drifting through areas with strong prevailing winds, especially when those winds oppose migration along the GCR, or when they coincide with ecological barriers, as these would be strong motivations to maximize local wind support. By contrast, we expect falcons to follow the GCR or to overcompensate for previous or anticipated displacements through supportive or weak prevailing winds. We specifically expect major course and performance changes at the ITF, which is located over the Sahel-Sudan climate zone, and which we expect to mark the southern limit of the desert barrier in both seasons. Based on previous studies in other colonies we also expect falcons to detour to stop-over areas in the Horn of Africa independently from wind, and to make a relatively direct return to their summer range, resulting in a shorter spring than autumn migration. Finally, we expect that wind support along the falcons' realized routes to be an important driver of daily, regional and seasonal performance, in addition to landscape differences in daily travel time budgets.

## Methods

### Tracking falcons

**Fig. 1:**
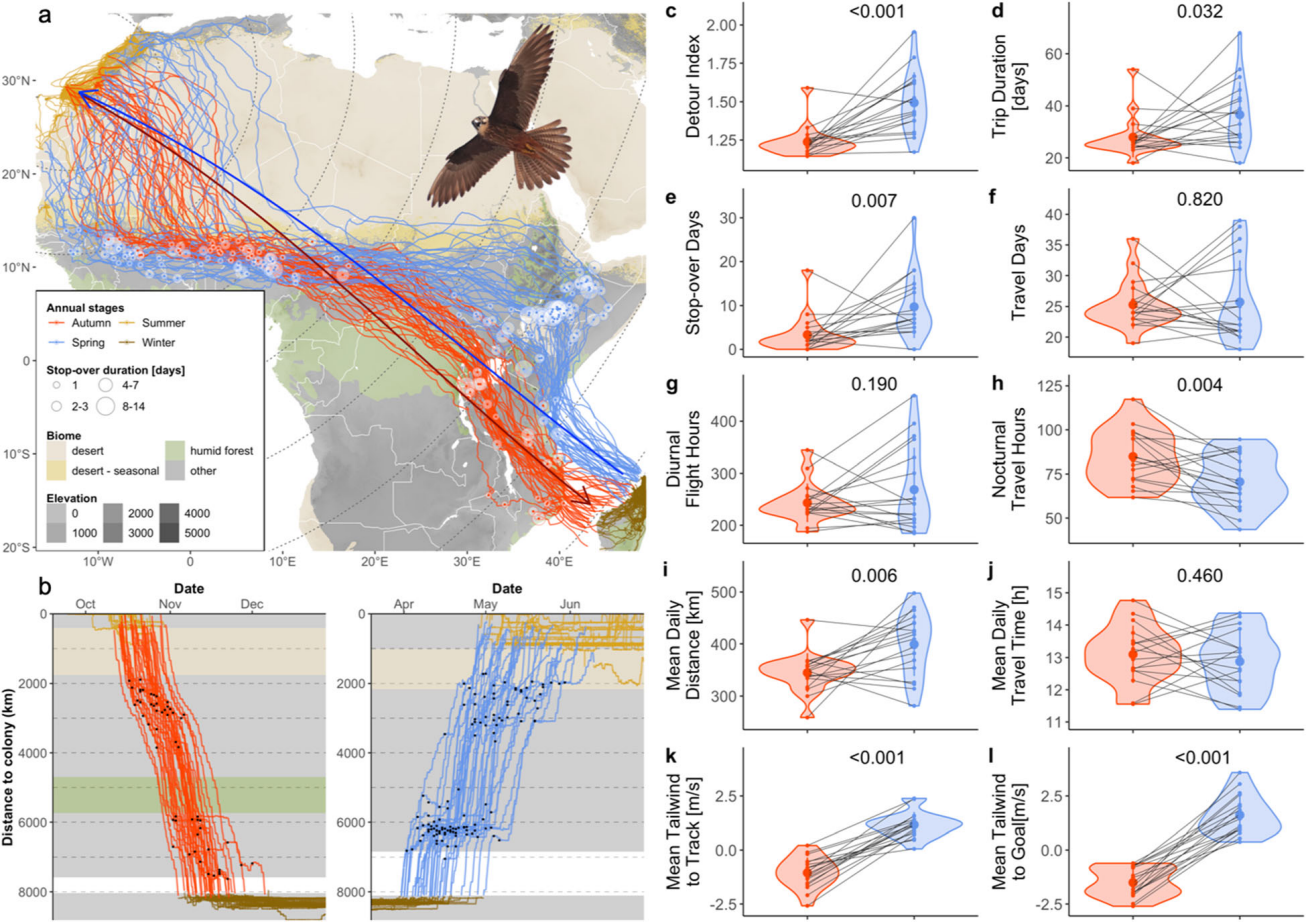
Autumn (*n* = 39) and spring (*n* = 36) **a** routes, **b** schedules, **c-l** performance metrics and **k-l** wind support metrics for 19 Canarian Eleonora’s Falcons travelling to and from their North Malagasy wintering grounds. **a** Migration routes and stop-overs (symbol legend) are shown in relation to supposed ecological barriers for migrant falcons: desert (beige = NDVI < 0.25 in both seasons, ochre = NDVI < 0.25 in spring only) and tropical rainforest (green). Other presumably hospitable habitats -mostly tropical savannahs- are shown in grey. Thick coloured lines indicate the shortest possible great-circle route from Alegranza to Madagascar in autumn (dark red), and from the falcons’ mean spring departure location to Alegranza in spring (dark blue). Dashed grey lines indicate distance to the colony at 1000 km intervals. **b** Timing of migration is shown as the increasing/decreasing distance to the breeding colony throughout each season. Black dots show stop-overs. **c-l** Seasonal performance and wind support metrics based on the first recorded trip of each individual in each season (*n* = 18). We show **c**, **d** detour extent and trip duration, **e**, **f** stop-over/travel days, **g**, **h** diurnal/nocturnal flight hours, **i**, **j** mean daily distances and travel time budgets, and **k**, **l** tailwind assistance with respect to the falcons’ realized travel direction and the great-circle direction to the seasonal destination. Large coloured dots and whiskers indicate the overall mean ± sd in each season (autumn = red; spring = blue). Black lines connect seasonal statistics (small dots) per individual. Labels show adjusted *p*-values from a pairwise t-test

During the breeding seasons of 2012, 2014, 2017 and 2018 a total of 40 Eleonora’s falcons were equipped with UvA-BiTS GPS-trackers (7.5 g) [35] on Alegranza islet (29°24′N, 13°30′W, 1050 ha, max 289 m a.s.l.). This is the northernmost islet of the Chinijo Archipelago in the Canary Islands, 160-300 km west off the African coast (Fig. 1a). The Eleonora’s falcon colony on Alegranza consists of an average of 127 breeding pairs, about 45% of the Canarian breeding population [36, 37], and is located at the western limit of the species’ breeding range.

Falcons were tagged with GPS attached as backpacks [38] using 6.35 mm wide Teflon harnesses. In total the device and harness weighed ~ 8 g, equivalent to 2.03–2.63% and 1.61–2.42% of the mass at capture for male (304 – 395 g) and female (330 – 495 g) falcons, respectively. During the non-breeding season, geographical positions were recorded at intervals ranging from minutes to hours (depending on solar-dependent battery power and different measurement schemes between night and day, and between migration and ‘wintering’ areas) with a horizontal precision of 3–15 m. Migration data were downloaded through a local antenna network every summer (July–October) between 2013 and 2020. We were able to download migration data for 19 individuals (10 males, 9 females, Table S1).

We are certain that at least 7 of the 21 individuals for which we were unable to retrieve data did return to the archipelago, but bred out of reach of the antenna network (i.e. on other islets, or shaded by steep cliffs). In addition, one tracker was detached soon after deployment and two others failed to communicate and did not download data. Such issues likely impaired data retrieval for several more individuals. These issues complicate assessment of tag effects. All considered, however, at least 72.5% of tagged birds returned to the Canary Islands at least once, and 13 falcons were tracked across two or more migrations (Table S1), and mostly successful breeding attempts, suggesting a limited impact of tagging on falcons’ migration and breeding performance.

### Defining migratory movements

To extract migration data, we defined the start and end of the wintering period as the first and last date a bird was recorded in Madagascar during each non-breeding cycle. The start and end of the breeding season are more difficult to define because Eleonora’s falcons engage in wide-ranging pre-breeding movements, scattering across staging areas up to hundreds of km’s from their breeding site [39]. Analogously, falcons often stopped-over in northwest Africa before initiating their autumn flight towards Madagascar. While such pre- and post-breeding periods form an integral part of avian migrations, and migratory fuelling strategies in particular [40], the focus of this study is on large-scale orientation behaviour and its consequences for realized wind support and travel performance. We thus excluded pre- and post-breeding movements for our analyses, taking the day on which falcons left the Western Sahara or Morocco as the start of autumn migration (Fig. 1a, b). We took the first stop-over day (i.e. days with < 100 km travelled between the first and last fix) in Western Sahara or Morocco, or the day on which falcons first reached the Atlantic Ocean, as the end date for spring migration. Two spring trips were only partially recorded and excluded from further analyses (B1014 in 2015, B2337 in 2018). In total, we retained 1,041,854 positions covering a total of 39 autumn and 36 spring migrations by 19 falcons (Table S1).

### Resampling and movement statistics

To couple movement data with global atmospheric reanalysis models [10, 41] we standardized the temporal resolution of the movement data to hourly intervals. Our resampling procedure allowed for deviations up to 10 min from an hourly interval. The resampled dataset comprised 49,314 locations covering 2294 bird days. Movement statistics (direction, step length, duration and speed) were calculated from each resampled fix to the next. To distinguish directed movements (i.e. travel) from localized foraging/resting we used an hourly speed threshold of 5 km h^− 1^. For each day we then calculated the total amount of travel time by summing the duration of all travel segments. Because falcons can migrate by night as well as by day (Fig. S1) we also determined the diurnal and nocturnal number of hours spent travelling, and calculated daily beeline distances (hereinafter: daily distances) as the great-circle (i.e. orthodromic) distance from the first to the last fix on each day. The mean daily travel speed was determined as the average trajectory speed across all travel segments (thus excluding intermittent foraging and resting events from travel speed calculations).

### Route segmentation: travel vs. stop-over days

Because Eleonora’s falcons are fly-forage migrants [31, 33] it is not easy to distinguish active travel from stop-overs. After inspecting frequency distributions of various movement statistics, we defined stop-over days as days on which birds achieved a daily beeline distance < 100 km. This classification is supported by marked differences in daily travel time budgets between rest and travel days (Fig. S1). According to our classification 39,694 locations were recorded on 1842 travel days, and 9620 locations on 452 stop-over days. We then segmented trips into periods of continuous travel and resting, and then calculated the total duration and mean longitude and latitude of each stop-over event (*n* = 175, duration: 1–14 days).

### Route annotation: biomes

Because falcons likely adjust orientation behaviour and performance to ecological barriers and foraging opportunities [28, 33] we distinguished between four biome categories, including two ‘hard’ ecological barriers (Sahara desert, Indian Ocean), a presumed ‘soft’ ecological barrier for falcons (i.e. the tropical humid forest of the Congo Basin) and lumping all other biomes (mostly tropical savannahs along the falcons’ routes) as ‘hospitable landscapes’ (Fig. 1a, b; Fig. S2). We included fixes over islands in sea-crossings. The boundaries of the tropical humid forest were extracted from a static global biome map [42] whereby we annotated flights over the Congo Basin as forest-crossing events (i.e. excluding flights over small forest patches in East Africa, Fig. S2). The barren ‘desert’ landscape of the Sahara is known to extend further south into the Sahel-Sudan zone during spring (start of wet season) compared to autumn (end of wet season). Therefore, we constructed Maximum-Pixel-Value composite maps of seasonal NDVI from the MODIS 16-day NDVI product (MCD43A4 V6, 500x500m) [43] in Google Earth Engine [44]. We thereby considered all NDVI data between the mean seasonal start and end date of migration for each year between 2012 and 2020 (Additional file 3). Pixels with a maximum seasonal NDVI < 0.25 were considered as ‘desert’, because it has been suggested that falcons avoid areas under this threshold [30]. We then annotated Sahara-crossings as the longest continuous desert-crossing on each trip (Fig. S2). Daily biome values were calculated as the modal biome value on each bird day.

### Route annotation: wind support and orientation

We used the RNCEP package [45] to annotate all GPS-fixes recorded on travel days with u- and v-wind components as estimated at the 850mb pressure level (~ 1170–1590 m asl) in the NOAA-NCEP Reanalysis II model [46], corresponding to a mean flight altitude of 1344 ± 880 m asl in our resampled dataset. Wind estimates were tri-linearly interpolated in time and space from 6-hourly model data with a horizontal resolution of 2.5° × 2.5°. From these wind components we derived wind speed and direction at each location, which we used to calculate the strength of tail−/headwinds and sidewinds relative to (i) the realized travel direction (i.e. direction from t_i_ to t_i + 1_) and (ii) to the shortest possible GCR from t_i_ to the seasonal goal destination. To simulate the shortest possible route we used the geosphere package [47] to determine the nearest point on the island of Madagascar from each point along the autumn route. We used the centre of Alegranza islet (29°24′N, 13°30′W) as the intended spring destination. To obtain daily estimates of wind support we averaged hourly tailwind and sidewind strength relative to the realized and great-circle travel direction across all travel segments on each travel day.

In order to determine when and where falcons improved tailwind assistance by deviating from the GCR we calculated a Local Wind Gain Index (LWGI) as:

$$ LWGI=\frac{W\ast \cos \left(\theta \right)-W\ast \cos \left(\beta \right)}{W} $$

whereby W represents total wind speed, *θ * represents the angle between the direction of the wind and the birds travel direction, and *β* represents the angle between the direction of the wind and the GCR to the seasonal destination. If LWGI = 0 then the bird is following the GCR (i.e. *θ* = *β*). If winds blow in the same direction this would be the optimal situation from an energy-minimizing perspective. If LWGI = 1 then a bird has gained the equivalent of the total prevailing wind speed by deviating from the GCR. This can happen, for example, if the wind blows perpendicular to the GCR while providing a pure tailwind along the birds’ chosen direction (i.e. *θ* = 0, *β* = 90°). The highest possible value of LWGI = 2, and occurs if the prevailing winds provide a pure tailwind in the chosen direction, but a pure headwind along the GCR (i.e. *θ* = 0, *β* = 180°). Conversely, − 2 < LGWI < 0 indicate situations where a bird experiences less wind support along its chosen route vs. the GCR.

We further classified orientation responses to sidewinds from the ratio between the falcons’ sideward displacement rate and the sidewind strength relative to the GCR, distinguishing between overcompensation (ratio < − 0.2), full compensation (− 0.2 < ratio < 0.2), partial compensation (0.2 < ratio < 0.8), full drift (0.8 < ratio < 1.2), overdrift (ratio > 1.2), and cases with no/weak sidewinds (sidewind strength < 0.5 ms^− 1^) [14, 15, 48]. Overcompensation is thus expected to compromise local wind support (i.e. LWGI < 0) compared to the GCR (i.e. compensation), whereas partial compensation and (over) drift are expected to result in local wind support gains (i.e. LGWI > 0).

### Route contextualisation: ITF, climate and wind fields

We obtained decadal positional estimates for the ITF from the NOAA Climate Prediction Center – West African Monsoon Monitoring project (https://www.cpc.ncep.noaa.gov/products/international/itf/itcz.shtml). This data product consists of latitudinal estimates of the ITF at intervals of 5° longitude between 10°W to 35°E, and from April through October each year (coinciding with the West African monsoon, but unfortunately do not include information for the second half of the autumn migration in November). The positional estimates are based on a subjective interpretation of (i) surface dewpoint temperature and (ii) lower-level (i.e. 925mb) wind fields by expert meteorologists. For visualization purposes we mapped the mean and inter-quartile latitude (Q25%-Q75%) of the ITF in October and April for autumn and spring, respectively.

Next, we used the RNCEP package [45] to visualize synoptic wind fields and seasonal rainfall during our study period. To do this we downloaded wind estimates relative to the 850 mb pressure level and precipitation rates relative to a gaussian grid from the NOAA-NCEP Reanalysis II model [46]. We downloaded data for the entire migratory domain (as defined by the min and max longitude and latitude recorded on migration) and for the months of April and October throughout the study period (autumn 2012 – spring 2020). To visualize prevailing atmospheric flows, we averaged u- and v-components for each node in the NCEP model grid across the entire study period. To visualize seasonal rainfall, we converted precipitation rate estimates (mm m^− 2^ s^− 1^) to total precipitation estimates (mm) for each six-hour interval in the NCEP model. We then integrated these values across each month, and finally averaged monthly rainfall estimates at each node in the NCEP-grid across the study period, for April and October respectively.

In addition, to visualize the timing of migratory movements in relation to the timing of ITF and climatic shifts, we superimposed falcon data on a Hovmöller diagram for mean daily rainfall across mainland Africa – a common approach to display ‘waves’ in meteorological data. To do this, RNCEP rainfall data were integrated to daily rainfall estimates at every node in the NCEP grid. For each day of the year we then averaged daily rainfall estimates across each band of 2.5° latitude and all years in the study period.

### Statistical analyses

#### Seasonal detours and performance patterns

We determined the extent of seasonal detours as the ratio of the cumulative distance travelled across all travel segments on travel days over the great-circle distance from the first to the last GPS-fix on each trip. Next, we calculated the total duration, number of stop-over and travel days, total number of nocturnal and diurnal travel hours, average daily distance, average daily travel time, average daily mean travel speed and average daily mean tailwind assistance (relative to the realized route and the GCR) for each trip. Using paired two-sided t-tests we then tested for seasonal differences in each of these performance and wind assistance metrics, using the first recorded trip for each individual in each season. These pairwise tests excluded one individual for which we lacked spring migration data (B2337, Table S1).

Next, we used generalized linear mixed regression models (GLMMs) to test if trip duration, rest/travel days and tailwinds were associated with detour extent, and whether this association differed between seasons (*n* = 75 trips). All response variables were fitted assuming a Gaussian error distribution and identity link function. The response variables trip duration, travel days and rest days were skewed positively and were log-transformed. For each response variable we compared models including additive and interaction effects of detour extent and season. We further allowed for random variation in intercepts between individuals and years to account for individual variability in behaviour and repeated sampling of the same individuals (number of trips recorded per season varied from 1 to 4 between individuals), and to account for repeated measurements within years. We calculated Aikaike’s Information Criterion corrected for small sample sizes (AICc) and AICc weights, and used the rsq package [49] to determine the coefficient of determination (R^2^) of each model and the partial R^2^ values for fixed and random terms, respectively, using the rsq package [49]. We then calculated deltaAICc as the difference in AICc values between each model and the model with the lowest AICc value. The best model for each response variable was identified as the most parsimonious model within those ranked with ∆AICc < 2 (further corroborated by AICc weights), after which the lmerTest package was used to obtain *p*-values for fixed effects using Satterthwaite’s method [50].

#### Route choice in relation to the ITF

We aimed to test the relationship between ITF latitudinal position and the latitude at which falcons crossed the Sahel-Sudan climate zone. We extracted migration data for that stage of the trip by selecting all fixes between 17.5°W - 37.5°E and between 5°N - 15°N on travel days where the daily travel direction was oriented due east in autumn (65° < dir < 125°) or due west in spring (− 125° > dir > − 65°). ITF latitudinal position estimates are provided every 10 days and for every five degrees longitude, and so we averaged the falcons’ latitudinal position across corresponding spatiotemporal blocks (*n* = 293). Finally, we used GLMMs to test the effects of the ITF latitudinal position, season, longitude, their additive and interaction effects on the latitude of trans-Sahelian movements, allowing for randomly varying intercepts between individuals. The most parsimonious model within those ranked with ∆AICc < 2 was considered the best model.

#### Daily performance vs. biome and wind conditions

To help determine candidate models for an exhaustive model selection procedure, we first explored seasonal relationships of daily performance with biome and wind assistance variables, and the extent of individual variation in daily performance metrics. We used GLMMs to test (1) how daily travel time, biome and season (fixed effects) affected daily distance and daily mean travel speed, and to test (2) how daily mean head−/tailwinds, biome and season (fixed effects) affected daily travel time, daily distances and daily mean hourly speeds (building separate models for head−/tailwinds along the realized route and the simulated GCR). The response variables daily distance and daily mean speed were positively skewed and log-transformed. All models included randomly varying intercepts between individuals and years. The large sample of travel days (*n* = 1842) allowed to use ∆AIC rather than ∆AICc to identify the most parsimonious models. Additionally, we calculated the full R^2^ of each model, and the partial R^2^ for fixed and random terms.

Based on insights from simple models, we constructed a larger set of GLMMs to better understand variation in daily distances and daily mean travel speeds. Candidate models included: head/tailwind and absolute sidewind strength experienced along the realized route, daily travel time, biome, season, their additive effects, interaction effects of wind variables with daily travel time, biome and season, and an interaction effect of daily travel time with biome and season as fixed effects.

All data analyses and visualization was conducted in R v.3.5.3 [51]. Graphs and maps were produced with ggplot2 [52].

## Results

### General route description

Eleonora’s falcons initiated their autumn migration from the Canary Islands in the second half of October and completed the trip during the first 3 weeks of November (Fig. 1a-b, Table S1). They departed in a southeast direction but usually changed course more southward at some point across the Sahara, and maintained this direction until deep into the savannahs of the Sahel-Sudan zone (Fig. 1a). There, falcons abruptly changed direction eastward in a relatively narrow migration corridor, making irregular stop-overs. When they reached the point where they would have completed the desert-crossing had they followed the GCR from Alegranza to Madagascar, some falcons changed course southeast and directly crossed the tropical rainforest of the Congo Basin roughly along the GCR. However, more commonly falcons continued over the Sahel for another 500–1500 km before reorienting southeast, skirting the northeast corner of the Congo Basin and reaching the East African savannahs between Lake Victoria and the northernmost point of Lake Tanganyika. There they maintained a more southward direction and made irregular stop-overs before finally orienting east-southeast across the Mozambique Channel near its narrowest 420 km point.

The falcons departed on spring migration in the first half of April and reached their pre-breeding sites mostly in the first half of May (Fig. 1b, Table S1). In spring, falcons displayed an even more pronounced zig-zag migration pattern than in autumn (Fig. 1a). Upon leaving northern Madagascar falcons roughly followed the GCR to Alegranza, during an 800–1500 km flight across the Indian Ocean. Upon reaching East Africa they detoured northward to stop-over sites that were clustered to the south and east of the Ethiopian Highlands, although some individuals took a more direct route, making stop-overs in Uganda and South Sudan. Thereafter, falcons did not return to Alegranza directly from East Africa, but instead travelled due west across the Sahel-Sudan zone for 2500–4000 km, postponing the desert-crossing far beyond the point where they would have reached desert if they had followed the GCR directly from Madagascar (Fig. 1a). Falcons then frequently stopped-over in the West African Sahel, in areas slightly further south than where they stop-over in autumn, before making the desert-crossing (Fig. 1a).

### Seasonal detours, performance and wind support

On average, Eleonora’s falcons travelled 1606 km more in spring (11,170 ± 1220 km) than in autumn (9564 ± 1220 km, Table S1). The seasonal detours thus equated to 1.44 and 1.23 times the length of the GCR between their start and end locations (~ 7800 km) in autumn and spring, respectively (Fig. 1a, c; Table S1). On average they needed 7 more days to complete their more detoured spring migration (34 ± 10 days) than their autumn migration (27 ± 6 days, Fig. 1d). However, this difference was largely due to falcons making about 6 more stop-over days in spring (9 ± 6 days) than in autumn (3 ± 3 days, Fig. 1e). Despite the large difference in cumulative travel distance between seasons, we found no significant difference in travel days between autumn (25 ± 3 days) and spring (24 ± 6 days, Fig. 1f). Males and females showed very similar patterns in overall performance within and between season (Table S2).

Falcons travelled for a similar amount of daylight hours in autumn (241 ± 34 h) and spring (250 ± 67 h, Fig. 1g), and slightly more hours at night during autumn (85 ± 13 h) than spring (71 ± 13 h, Fig. 1h). However, on average, there was no significant difference in daily travel time budgets between seasons (autumn: 13 ± 1 h, spring: 13 ± 2 h) (Fig. 1j). Falcons did achieve significantly greater daily distances during spring (412 ± 64 km) than during autumn (352 ± 36 km) (Fig. 1i), which was associated with a large seasonal difference in mean daily tailwind assistance: falcons generally experienced headwinds along their autumn routes (− 1.1 ± 0.7 ms^− 1^) while benefiting from tailwinds along their spring routes (1.1 ms^− 1^) (Fig. 1k). There was an even greater seasonal difference in wind support along the GCR, with autumn headwinds (− 1.6 ± 0.7 ms^− 1^) and spring tailwinds (1.7 ± 0.9 ms^− 1^) being more pronounced along the GCR than along the realized route (Fig. 1k-l).

Plotting linear relationships suggested a strong correlation of mean individual trip duration, stop-over days and travel days with detour extent per season, but not so for realized tailwind support (Fig. S3). GLMMs across all trips (*n* = 75) confirmed that seasonal differences in overall trip duration are accounted for by seasonal differences in detour extent (Table S3). However, variation in stop-over and travel days was best explained by the additive effect of season and detour extent (Fig. S3, Table S3). That is: rest days and travel days both significantly increased with detour extent, but falcons made significantly more stop-over days and needed fewer travel days to complete detours of comparable extent in spring than in autumn. Furthermore, mixed models showed that very little variation in trip duration and travel days could be attributed to individual or year differences (Table S3). However, random effects did account for ~ 12% of variation in stop-over days (Table S3), and detailed regression outputs showed this effect was driven by individual differences in stop-over time budgets during spring migration.

Although we did not find a significant correlation of realized tailwind with detour extent based on average individual metrics (Fig. S3, Table S3) the best model based on all trips does suggest a negative interaction effect of season and detour extent on realized tailwinds (Tables S3-S4). However, this interaction explains little variation in addition to the 75% of variation already explained by a model including only season as fixed predictor (Table S3). The partial R^2^ for random model terms further showed that variability in wind support was not driven by individual or year differences (Table S3).

### Realized wind support and orientation through seasonal climates and wind fields

**Fig. 2:**
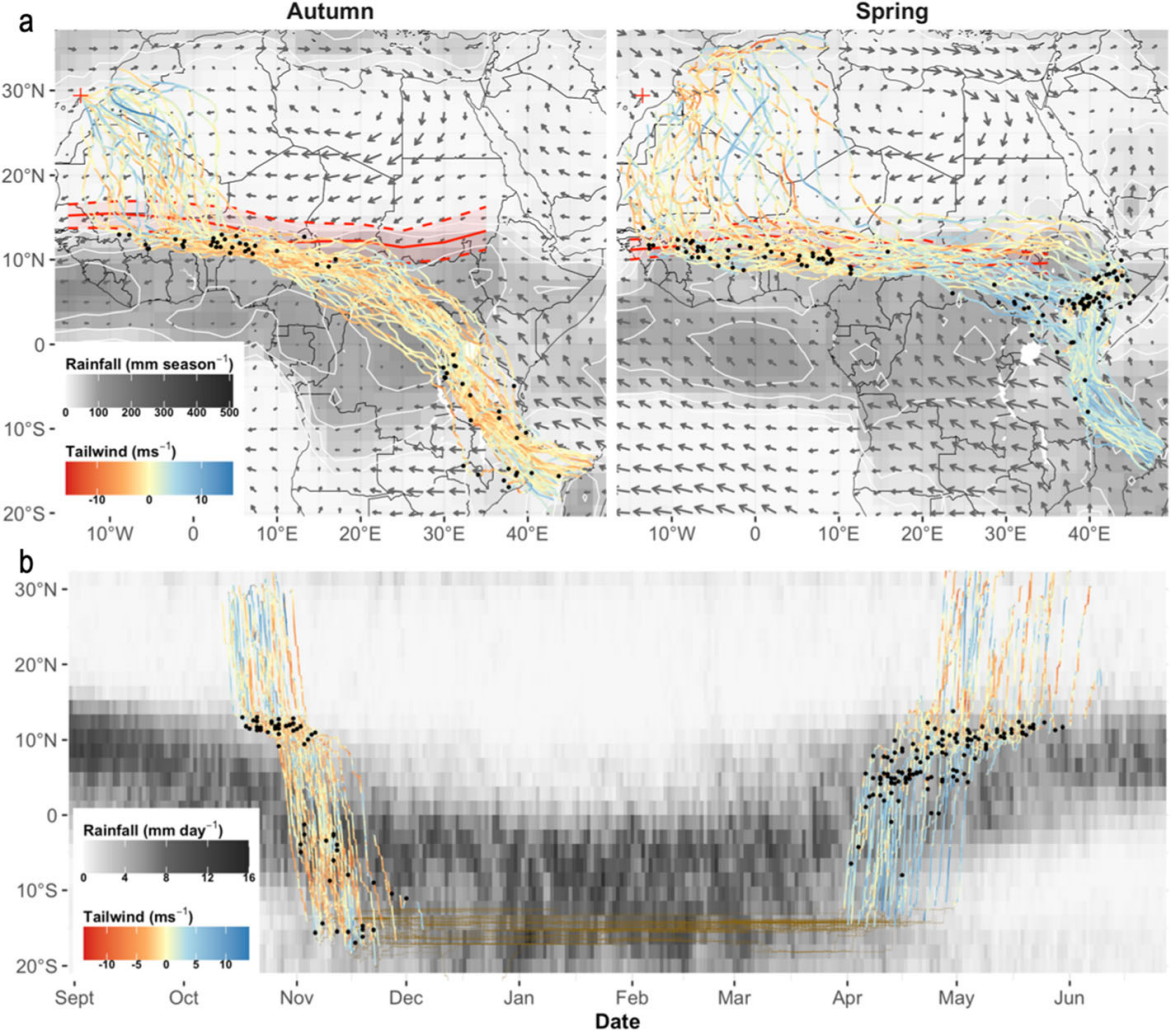
Eleonora’s falcon migration routes and timing relative to the seasonal position of the ITF and African rainfall. **a** Tracks are coloured according to tailwind strength along the falcons’ realized travel direction (reds = headwinds, blues = tailwinds). Red ribbons show the mean latitudinal position (solid line) and Q10%-Q90% latitudinal range (dashed lines) of the ITF at each 5° longitude. Note that ITF positions and rainfall heatmaps are based on data for October and April, which is representative for the period in which falcons initiate eastward/westward movements along the ITF in autumn/spring, respectively. The ITF would have shifted further south/north by the time falcons crossed the Congo Basin in autumn and by the time they reached West Africa in spring, respectively. **b** Falcon tracks superimposed on a Hovmöller diagram for mean daily rainfall at every 2.5° latitude across mainland Africa, visualizing the seasonal shift in rainfall latitude across Africa associated to the shifting position of the ITF

**Fig. 3:**
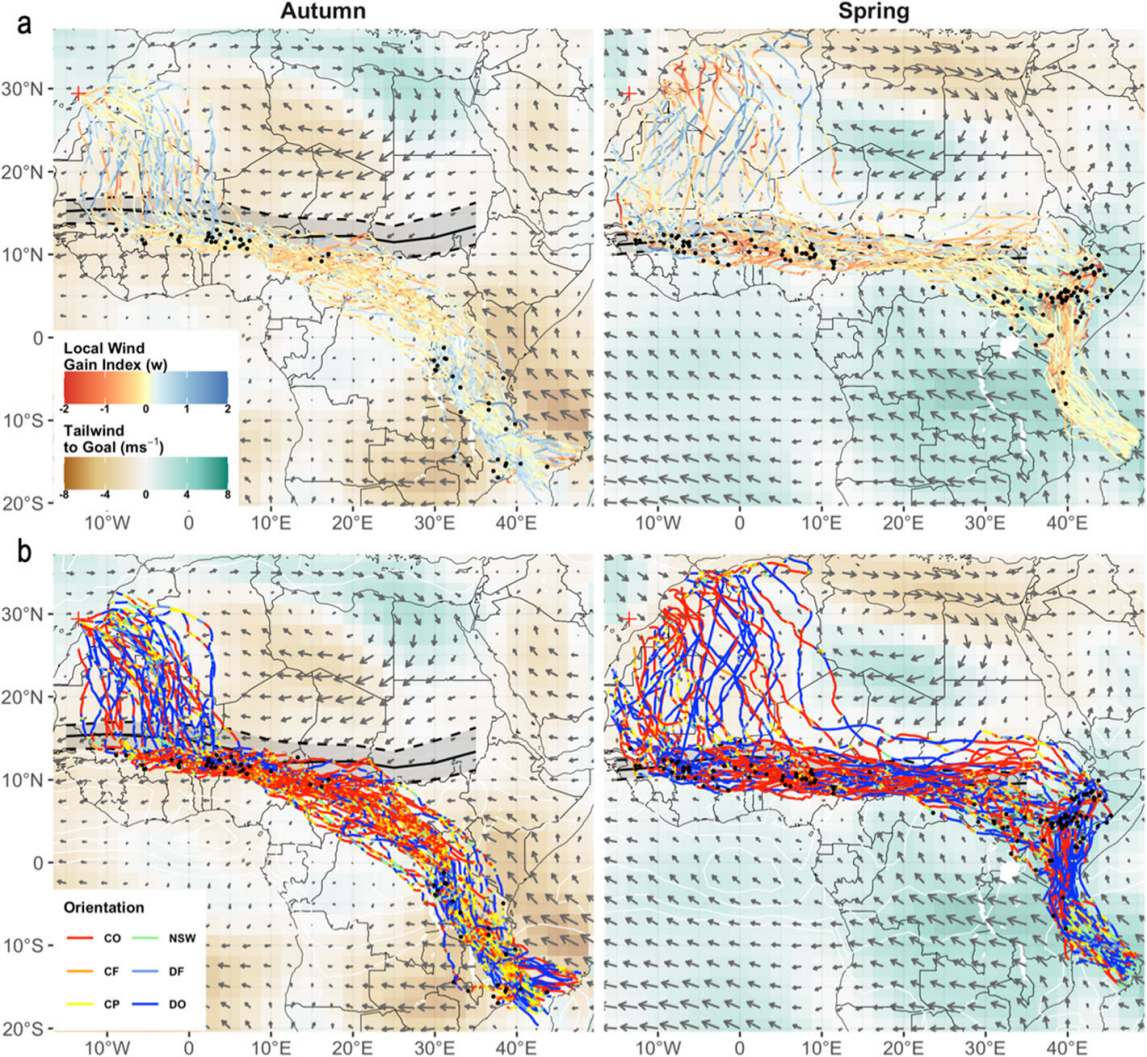
Wind support gains and orientation responses of Eleonora’s falcons to sidewinds through Africa's prevailing wind regimes. Black ribbons show the seasonal position of the ITF (cf. red ribbons Fig. 2). The heatmap indicates mean wind support along the GCR to the seasonal destination from each node in the wind data (browns = opposing winds, greens = supportive winds, white = perpendicular winds). We determined whether the tailwinds experienced by falcons along their detours represent (**a**) gains/losses in wind support compared to what was available along the GCR (reds = losses, blues = gains). **b** Orientation responses were classified as over-drift (DO, i.e. heading downwind from the GCR), full drift (DF), partial (CP) or full compensatation (CF, i.e. staying on the GCR through sidewinds), overcompensation (CO, i.e. heading upwind the GCR). Cases where falcons stayed on the GCR in absence of sidewinds were not classified (NSW)

Large-scale changes in orientation behaviour across Africa’s biomes were associated with strong changes in seasonal climate and prevailing wind fields (Figs. 2-3, Fig. S4). In autumn, falcons experienced weak to moderately supportive tailwinds upon departure, but often also moderate headwinds further over the Sahara, until they crossed the ITF (Fig. 2a: blue line colour indicates supportive tailwinds along the realized track). Nevertheless, the southward course change over the Sahara was due to falcons overdrifting (Fig. 3b, Fig. S4), and thus associated with a substantial gain in wind support (i.e. a reduction in headwinds) through the GCR-opposing winds north of the ITF (Fig. 3a: blue tracks indicate gains in wind support). After the abrupt eastward course change (and stop-overs) south of the ITF, falcons overcompensated to track the northern front of the African rainbelt (Fig. 2a, b) and experienced moderate headwinds in doing so (Fig. 2a). Their tortuous fly-forage migration yielded a more irregular picture of wind gains/losses, but they incurred clear and increasingly large losses in wind support by continuing east of the GCR, and further on while crossing the tropical rainforest belt (Fig. 3a). That said, in absolute terms they faced only weak headwinds in these regions compared to what they would have experienced by tracking the GCR over the desert (Fig. 2a). After crossing the equatorial rain forest the falcons entered stronger, opposing wind fields once again, and realized a substantial headwind reduction by overdrifting and partially compensating for sidewinds across East Africa (Fig. 3b, Fig. S4). They finally crossed the Mozambique Channel without strong wind support -sometimes even headwinds (Fig. 3a)-, but still improved wind support compared to the GCR by (over) drifting in many cases (Fig. 3b).

In spring, the direct ocean-crossing to East Africa was associated with strong tailwind support due to prevailing south-easterlies (Fig. 2a). Note that this translated to a near-zero wind gain due to falcons’ travel direction being closely aligned with the GCR here (note category NSW in Fig. 3a, b; Fig. S4). Nevertheless, relatively slight deviations from the GCR are often classified as overdrift over the Indian Ocean because winds are particularly strong there, and so our 0.5 ms^− 1^ threshold for “no sidewinds” is frequently exceeded this region (Fig. 3b). The falcons continued to enjoy strong tailwind support as they detoured northward across mainland Africa (Fig. 2a), but in so doing they did not take full advantage of the available wind support along the GCR (Fig. 3b). In fact, for most of the overland spring migration falcons displayed a mixed pattern of both overdrift and overcompensation behaviours (Fig. 3b, Fig. S4).

Falcons that stopped-over in eastern Ethiopia and Somalia tended to depart in a northwest direction towards the desert, thereby overshooting the ITF, and then reoriented west-southwest in face of strong sidewinds over the eastern Sahara. All falcons travelled due west for at least several thousand km in a relatively narrow corridor that overlapped with the average position of the ITF during our study period (Fig. 2a), and stopping well south of the northern rain front in spring (Fig. 2b). They seemingly postponed the desert-crossing until West Africa, thereby tolerating a relatively large loss in local wind support compared to a more direct return (Fig. 3a). However, at worst this translates to a weak headwind near the ITF (Fig. 2a). Realized tailwinds over the desert appear highly variable (Fig. 2a) but falcons generally reduced headwind resistance by detouring from the GCR across the Sahara (Fig. 3b) and endured strong headwinds mostly at the final stages of the desert-crossing (Fig. 2a) as they overcompensated for sidewinds towards their pre-breeding sites (Fig. 3b).

### Route choice along the ITF

**Fig. 4:**
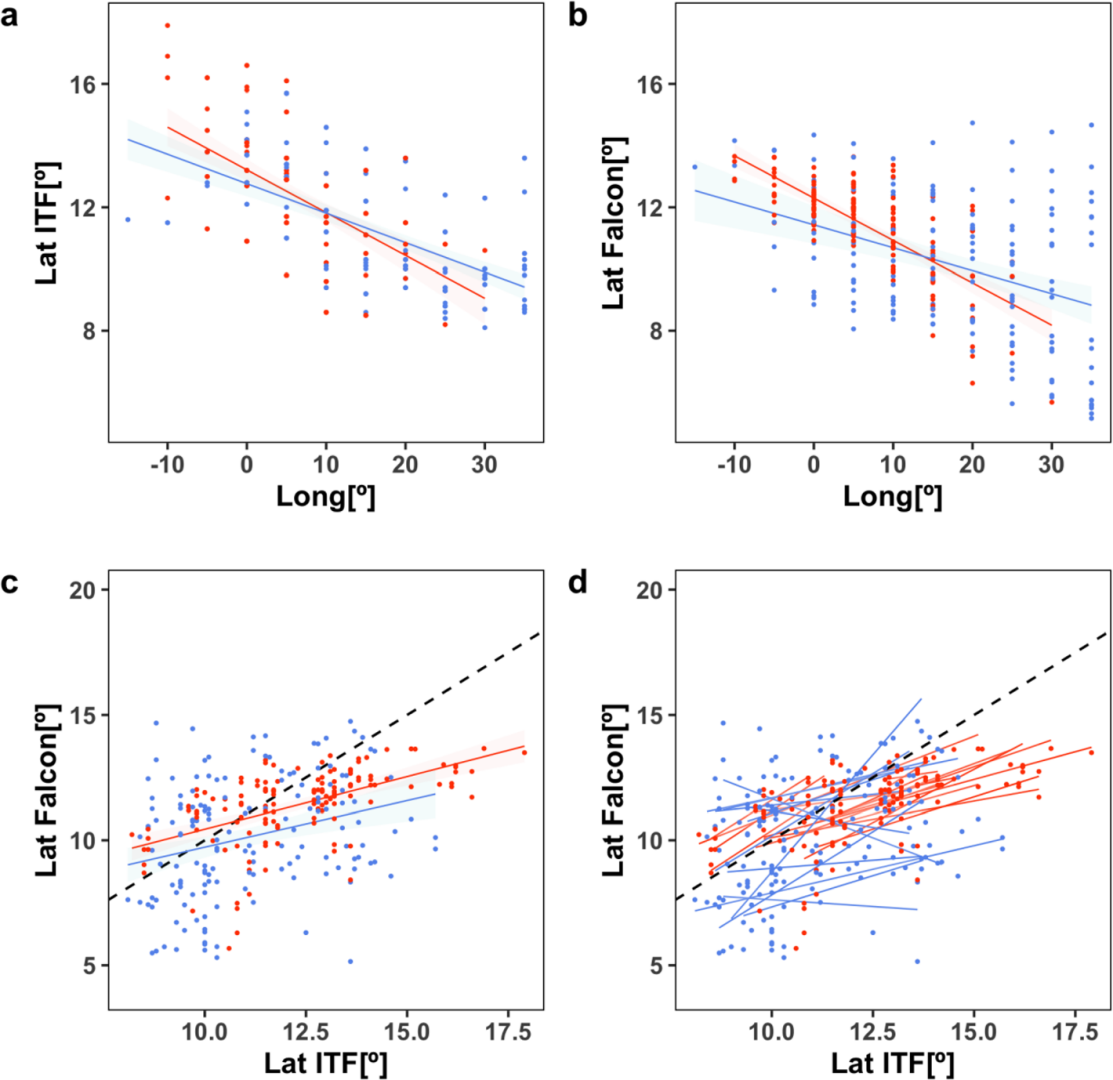
Seasonal linear relationships between the latitude of trans-Sahelian movements, longitude and the ITF position (autumn = red; spring = blue). **a**, **b** Latitude of the ITF and the trans-Sahelian migrations of falcons in relation to longitude. **c** Latitude of the trans-Sahelian migrations in relation to the latitude of the ITF at corresponding longitudes and times. The black dotted line indicates where latitude of falcons and ITF would match. Points below the black line correspond to cases where a falcon travelled south of the ITF. **d** Individual trend lines for each season showing consistency of the linear correlation between falcon and ITF positions

In general, the latitudinal position of both the ITF and the falcons decreased significantly with longitude in both seasons (Fig. 4a-b), although the migration corridor overlapped with the ITF much more closely in spring (Fig. 2). The latitude at which falcons travelled across the Sudan-Sahel zone was significantly and positively correlated with the latitudinal position of the ITF across the full dataset (Fig. 4c), but the effect was more consistent among individuals in autumn than in spring (Fig. 4d). GLMMs showed that ITF could not fully account for the longitudinal decrease in the falcons’ latitudinal position, but the ITF position competes with season as a significant predictor variable (Tables 1 and 2). Random individual differences accounted for a similar amount of variation in the latitude of trans-Sahelian migrations as ITF position (Table 2).

**Table 1:** Selecting the best GLMM for the latitude of trans-Sahelian migration

Model (fixed effects)	df	AIC	∆AIC	AIC Weight	R2 full	R2 fixed	R2 random
~ long*ITF*season	10	1086	0	1	0.52	0.32	0.20
~ long*season	6	1096	11	0	0.48	0.32	0.17
~ ITF + long	5	1106	21	0	0.47	0.31	0.16
~ long*ITF	6	1108	22	0	0.47	0.31	0.16
~ long	4	1116	31	0	0.44	0.30	0.14
~ long+season	5	1117	31	0	0.44	0.31	0.14
~ ITF*season	6	1139	54	0	0.42	0.21	0.21
~ ITF + season	5	1141	56	0	0.41	0.22	0.19
~ ITF	4	1156	70	0	0.37	0.18	0.19
~ season	4	1214	129	0	0.23	0.09	0.13
~	3	1245	160	0	0.14	0.00	0.14

Models included fixed effects for the latitudinal position of the ITF, longitude, season, their additive effects and interaction effects. We also allowed for intercepts to vary randomly between individuals in all models. Models are ranked according to increasing ∆AIC/decreasing AIC weight, with the best performing model on top

**Table 2:** Model coefficients as estimated by the best GLMM for the latitude of trans-Sahelian migration

Model term	Estimate	Std. Error	df	t value	Pr(>|t|)
**(Intercept)**	**9.530**	**1.173**	**292.895**	**8.124**	**< 0.001**
**lat_ITF**	**0.228**	**0.087**	**290.042**	**2.637**	**0.009**
**long**	**−0.345**	**0.093**	**282.856**	**−3.692**	**< 0.001**
spring	1.679	2.028	282.828	0.828	0.409
**lat_ITF:long**	**0.018**	**0.008**	**284.540**	**2.389**	**0.018**
lat_ITF:spring	−0.232	0.159	283.446	−1.463	0.145
long:spring	0.217	0.119	281.549	1.828	0.069
lat_ITF:long:spring	−0.012	0.010	282.362	−1.257	0.210

We consider coefficient estimates to be significant at *P* < 0.05 (bold)

### Wind and biome effects on migratory performance

Within seasons, daily travel distances peaked and waned across different geographical regions (Figs. S5-S6), which was largely due to differences in daily travel time budgets between biomes (Fig. S2). We found particularly long travel days over the desert in both seasons, over the sea in spring, and to a lesser extent over the tropical rainforest in autumn (S5-S6). Exploratory analyses further revealed differences in daily mean speed between biomes, with particularly fast travel over the sea in both seasons, and to a lesser extent over the desert (Fig. S6). However, realized tailwinds were higher over the desert than over the sea in autumn, and falcons generally enjoyed weaker tailwinds over the desert than over other biomes in spring (Fig. S6).

**Fig. 5:**
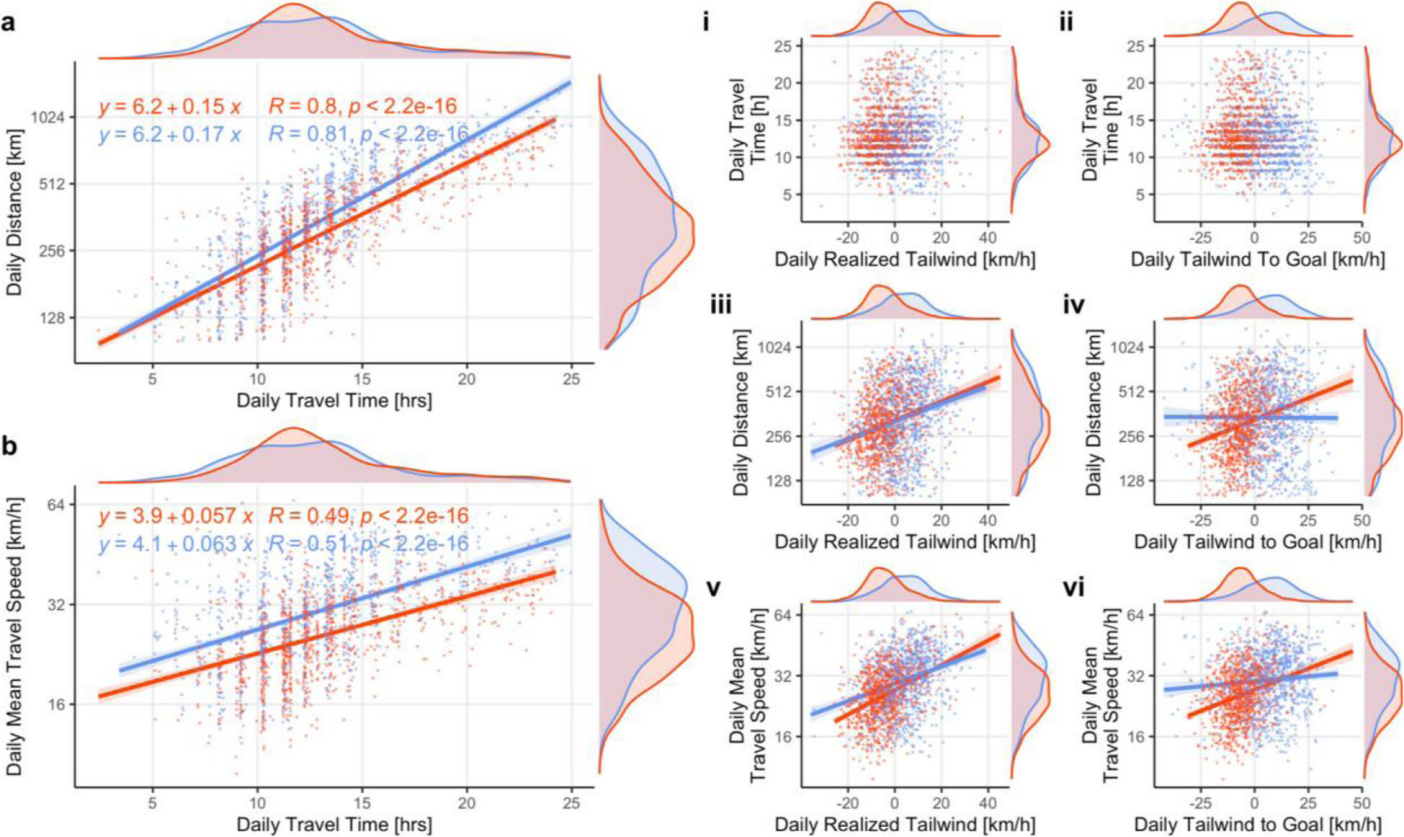
Exploring linear relationships between daily migration parameters and realized/available wind support (*n* = 1842 travel days). Linear relationships of **a** daily distance and **b** daily mean travel speed with daily travel time budgets. Linear relationships of (i-ii) daily travel time budgets , (iii, iv) daily distances and (v, vi) daily mean travel speed with (i, iii, v) realized wind support and (ii, iv, vi) wind support along the GCR during autumn (red) and spring (blue) migration. Y-axes for daily distances and daily mean travel speeds were log-transformed. Regression lines are estimated according a linear model (y ~ a + b*x) for each season (autumn = red; spring = blue), and are only plotted if there was a significant (*P* < 0.05) effect of x in at least one season

Plotting linear relationships showed a highly positive correlation of daily distance with travel time, and of daily mean travel speeds with travel time, in both seasons (Fig. 5a-b). Both daily distance and daily mean travel speeds increased more strongly with travel time in spring than in autumn (Fig. 5a-b: note greater estimates for slopes in spring) and daily mean speeds were greater in spring (Fig. 5b: note greater intercept estimate in spring).

Daily travel time itself was best explained by biome differences and not driven by realized tailwind (Table S5, but note very low R^2^ of biome model). By contrast, daily distance and daily mean travel speed were both positively correlated with daily realized tailwind (Fig. 5iii, v; Table S5). Our best models included additive effects of realized tailwind and biome on daily distance, and interaction effects of realized tailwind, biome and season on daily mean travel speed (Table S5). Analogous models using tailwinds along the GCR instead of realized tailwinds as a predictor showed a similarly positive wind effect on daily distance and daily mean travel speed during autumn, but not so during spring migration (Fig. 5iv,vi; Table S6). Random individual differences did not account for substantial variation in any of the response variables.

Our final model selection procedure revealed that daily distance (Table 3) and daily mean travel speeds (Table S7) were best explained by models including interaction effects of daily travel time and biome with realized tailwind and absolute realized sidewind strength. These models accounted for > 71% of variation in daily distance (Table 3), > 45% in daily mean travel speed (Table S7), with no remaining seasonal differences in these performance metrics. Detailed outputs for these models (Table 4) revealed significant positive effects of realized tailwind and daily travel time, and a negative effect of the biome ‘other’ and a positive effect of ‘sea’ (with ‘desert’ being the reference group for estimating the intercept), and biome-specific effects of travel time on both distance and speed. In addition, we found biome-specific effects of realized tailwinds on daily mean travel speeds, and no evidence for random individual or year differences in daily distance nor travel speed.

**Table 3:** Selecting the best GLMM for log-transformed daily distance (*n* = 1842 travel days)

Model (fixed effects)	df	AIC	∆AIC	AIC Weight	R2 full	R2 fixed	R2 random
~ (tailwind_track + abs (sidewind_track)) * travel_hrs * biome	***27***	***758.48***	***0.00***	***1.00***	***0.71***	***0.70***	***0.00***
~ (tailwind_track + abs (sidewind_track)) * biome + season	12	801.46	42.98	0.00	0.20	0.19	0.01
~ biome * travel_hrs	9	817.81	59.33	0.00	0.68	0.67	0.01
~ (tailwind_track + abs (sidewind_track)) * travel_hrs + biome	15	849.94	91.46	0.00	0.70	0.69	0.00
~ travel_hrs * abs (sidewind_track)	10	859.93	101.45	0.00	0.65	0.63	0.01
~ tailwind_track * travel_hrs	9	878.35	119.87	0.00	0.68	0.67	0.01
~ abs (sidewind_track) + travel_hrs	7	879.77	121.29	0.00	0.65	0.63	0.01
~ biome + abs (sidewind_track)	7	895.05	136.57	0.00	0.14	0.13	0.01
~ season + abs (sidewind_track)	6	908.30	149.82	0.00	0.03	0.02	0.01
~ season + biome + travel_hrs	7	909.63	151.15	0.00	0.67	0.67	0.00
~ season * tailwind_track	9	930.52	172.04	0.00	0.08	0.07	0.01
~ (tailwind_track + abs (sidewind_track)) * season	11	943.53	185.05	0.00	0.09	0.08	0.01
~ biome + tailwind_track + abs (sidewind_track)	8	992.51	234.03	0.00	0.18	0.18	0.01
~ tailwind_track + abs (sidewind_track)	7	1028.47	269.99	0.00	0.09	0.08	0.01
~ season + tailwind_track + biome	7	1045.48	287.00	0.00	0.18	0.17	0.01
~ season + tailwind_track	6	1055.16	296.68	0.00	0.08	0.07	0.01
~ season + abs (sidewind_track) + travel_hrs	7	1105.61	347.12	0.00	0.66	0.65	0.00
~ biome + travel_hrs	6	1110.26	351.78	0.00	0.67	0.66	0.01
~ abs (sidewind_track)	5	1125.04	366.56	0.00	0.02	0.01	0.01
~ (tailwind_track + abs (sidewind_track)) * travel_hrs * season	24	2575.59	1817.11	0.00	0.69	0.69	0.00
~ (tailwind_track + abs (sidewind_track)) * travel_hrs + season	15	2586.38	1827.90	0.00	0.69	0.68	0.00
~ (tailwind_track + abs (sidewind_track)) * biome * season	16	2587.98	1829.50	0.00	0.21	0.20	0.01
~ biome * tailwind_track	9	2611.55	1853.07	0.00	0.18	0.17	0.01
~ travel_hrs + tailwind_track + abs (sidewind_track)	8	2619.54	1861.06	0.00	0.68	0.68	0.01
~ (tailwind_track + abs (sidewind_track)) * biome	11	2619.68	1861.20	0.00	0.20	0.19	0.01
~ season * travel_hrs	9	2621.50	1863.02	0.00	0.65	0.65	0.00
~ (tailwind_track + abs (sidewind_track)) * travel_hrs	11	2685.52	1927.04	0.00	0.68	0.68	0.01
~ season * abs (sidewind_track)	9	2691.59	1933.11	0.00	0.03	0.02	0.01
~ tailwind_track * abs (sidewind_track)	10	2695.00	1936.52	0.00	0.09	0.08	0.01
~ biome + tailwind_track + travel_hrs	8	2697.32	1938.84	0.00	0.69	0.69	0.00
~ season * biome	8	2704.51	1946.03	0.00	0.15	0.14	0.01
~ biome + tailwind_track	7	2707.39	1948.91	0.00	0.18	0.17	0.01
~ season + travel_hrs	6	2808.74	2050.26	0.00	0.65	0.65	0.00
~ season + biome + abs (sidewind_track)	7	2809.29	2050.81	0.00	0.15	0.14	0.01
~ tailwind_track + travel_hrs	7	2810.58	2052.10	0.00	0.68	0.67	0.01
~ biome * abs (sidewind_track)	9	2810.77	2052.29	0.00	0.15	0.15	0.01
~ biome	5	2832.09	2073.61	0.00	0.14	0.13	0.01
~ travel_hrs	6	2834.03	2075.55	0.00	0.64	0.63	0.01
~ season + tailwind_track + travel_hrs	7	2835.69	2077.21	0.00	0.68	0.68	0.00
~ season + tailwind_track + abs (sidewind_track)	7	2928.90	2170.42	0.00	0.09	0.08	0.01
~ season + biome	6	2932.39	2173.91	0.00	0.14	0.14	0.01
~ tailwind_track	5	2950.18	2191.70	0.00	0.08	0.07	0.01
~ season	5	2951.18	2192.70	0.00	0.02	0.01	0.01
~ (1 | dev) + (1 | yr)	4	2963.73	2205.25	0.00	0.01	0.00	0.01

We tested an exhaustive set of models including fixed effects of tailwind and sidewind along the realized travel direction, daily travel time, season, their additive effects, and interaction effects between wind variables and daily travel time, biome and season. We further allowed intercepts to vary randomly between individuals and between years. Models are ranked according to increasing ∆AIC values, with the best performing model on top

**Table 4:** Fixed effects on log-transformed daily distance and daily mean travel speed as estimated by our best GLMMs

Response variable	Model term	Estimate	Std. Error	z value	Pr(>|z|)
**Log (Daily Distance)**	**(Intercept)**	**4.628**	**0.121**	**38.228**	**0.000**
	**tailwind_track**	**0.064**	**0.027**	**2.349**	**0.019**
	abs (sidewind_track)	0.023	0.032	0.733	0.463
	**travel_hrs**	**0.099**	**0.008**	**12.406**	**0.000**
	biome_humid forest	−0.165	0.274	−0.602	0.547
	**biome_other**	**−0.509**	**0.131**	**−3.875**	**0.000**
	**biome_sea**	**0.481**	**0.180**	**2.678**	**0.007**
	tailwind_track: travel_hrs	−0.002	0.002	−0.830	0.407
	abs (sidewind_track): travel_hrs	−0.001	0.002	−0.554	0.580
	tailwind_track: biome_humid forest	−0.167	0.108	−1.543	0.123
	tailwind_track: biome_other	−0.037	0.029	−1.260	0.208
	tailwind_track: biome_sea	−0.053	0.035	−1.498	0.134
	abs (sidewind_track): biome_humid forest	−0.017	0.089	−0.186	0.852
	abs (sidewind_track): biome_other	0.030	0.036	0.829	0.407
	abs (sidewind_track): biome_sea	−0.065	0.049	−1.325	0.185
	travel_hrs: biome_humid forest	0.003	0.019	0.148	0.882
	**travel_hrs: biome_other**	**0.024**	**0.009**	**2.637**	**0.008**
	**travel_hrs: biome_sea**	**−0.029**	**0.012**	**−2.439**	**0.015**
	tailwind_track: travel_hrs: biome_humid forest	0.008	0.007	1.053	0.293
	tailwind_track: travel_hrs: biome_other	0.002	0.002	1.217	0.224
	tailwind_track: travel_hrs: biome_sea	0.002	0.002	0.958	0.338
	abs (sidewind_track): travel_hrs: biome_humid forest	0.001	0.006	0.084	0.933
	abs (sidewind_track): travel_hrs: biome_other	−0.002	0.003	−0.641	0.521
	abs (sidewind_track): travel_hrs: biome_sea	0.005	0.004	1.319	0.187
**Log (Daily Mean Travel Speed)**	**(Intercept)**	**1.820**	**0.101**	**18.014**	**0.000**
	**tailwind_track**	**0.068**	**0.023**	**3.021**	**0.003**
	abs (sidewind_track)	−0.011	0.026	−0.402	0.688
	**travel_hrs**	**0.029**	**0.007**	**4.312**	**0.000**
	biome_humid forest	−0.159	0.228	−0.696	0.487
	**biome_other**	**−0.440**	**0.109**	**−4.020**	**0.000**
	**biome_sea**	**0.428**	**0.150**	**2.863**	**0.004**
	tailwind_track: travel_hrs	−0.002	0.002	−1.328	0.184
	abs (sidewind_track): travel_hrs	0.000	0.002	0.263	0.792
	**tailwind_track: biome_humid forest**	**−0.158**	**0.090**	**−1.748**	**0.081**
	tailwind_track: biome_other	−0.029	0.024	−1.204	0.229
	**tailwind_track: biome_sea**	**−0.051**	**0.029**	**−1.759**	**0.079**
	abs (sidewind_track): biome_humid forest	−0.014	0.074	−0.183	0.855
	abs (sidewind_track): biome_other	0.045	0.030	1.512	0.131
	abs (sidewind_track): biome_sea	0.023	0.041	0.562	0.574
	travel_hrs: biome_humid forest	0.003	0.016	0.169	0.866
	travel_hrs: biome_other	0.020	0.007	2.623	0.009
	**travel_hrs: biome_sea**	**−0.024**	**0.010**	**−2.436**	**0.015**
	tailwind_track: travel_hrs: biome_humid forest	0.008	0.006	1.359	0.174
	tailwind_track: travel_hrs: biome_other	0.002	0.002	1.281	0.200
	tailwind_track: travel_hrs: biome_sea	0.002	0.002	1.275	0.202
	abs (sidewind_track): travel_hrs: biome_humid forest	0.000	0.005	0.087	0.930
	abs (sidewind_track): travel_hrs: biome_other	−0.003	0.002	−1.313	0.189
	abs (sidewind_track): travel_hrs: biome_sea	− 0.001	0.003	− 0.312	0.755

We consider coefficient estimates to be significant at *P* < 0.05 (bold)

## Discussion

The latitudinal shift of the ITF and its associated wind fields is one of the most defining features of the Africa’s climate. Here, we show that wind support experienced by a trans-African migrant falcon plays a key role in shaping their daily, regional and seasonal performance, in addition to wind-independent adjustments in daily travel time. Falcons thereby tend to improve wind support by drifting through strong adverse wind fields, while compensating for displacements through relatively weak wind fields. They tolerated losses in wind support by detouring from the GCR in areas with weak or highly supportive prevailing winds. In contrast to Mediterranean colonies, spring migrations to the Canarian breeding grounds were much longer in length and substantially longer in overall duration than the autumn migration [27, 30]. However, as in other migrant birds where slower spring than autumn migrations have been reported, the added migration time was mainly due to the birds making a greater number of stop-overs during spring migration [53–55]. Greater tailwind support in spring meant Canarian falcons could actually cover their longer spring detours in the same number of travel days as the shorter autumn route. That said, the greater realized wind support in spring was due to the prevailing winds over Africa being generally more supportive for westward than eastward migration. In fact, falcons compromised wind support during spring detours over hospitable landscapes, whereas in autumn they avoided strong headwinds by detouring. Furthermore, we only found evidence of individual variation in performance parameters in spring (i.e. stop-over days), and falcons also showed more individual variation in route choice in this season. This suggests a stronger environmental canalization of route choice/development in opposing autumn wind regimes, whereas supportive spring wind fields allow for diversification of individual migration routines.

As in other fly-forage migrants, including several falcon species, we found that flexible travel time budgets are the main determinant of daily travel distances [56, 57]. We further show that daily travel time budgets were largely independent of wind support [58], and mainly varied due to falcons extending travel time over barriers (incl. nocturnal travel) and reducing travel time over hospitable habitats [28, 31]. Indeed, Eleonora’s falcons sometimes had to confront adverse winds while making ‘fast’ non-stop flights across ecological barriers (e.g. across the Mozambique Channel) and other times travelled few hours per day in supportive winds over hospitable landscapes (e.g. East Africa in spring). Despite regional variations in travel time budgets, realized tailwinds had a strong positive effect on daily mean travel speeds in both seasons, and models accounting for the combined effect of travel time and tailwind accounted for more than 60% of variation in daily travel distances, whereby the seasonal difference in realized wind support explains the marked seasonal difference in travel performance. Therefore, the faster spring travel is not evidence of a time-minimizing strategy, but rather a result of favourable seasonal conditions [59], whereby the late breeding and long pre-breeding period of Eleonora’s falcons likely buffers selection for time-minimization and early arrival to the summer range [36, 39].

As expected, the crossing of the ITF marked the end of the ‘desert’-crossing, and like individuals from other colonies, Canarian Eleonora’s falcons tended to make short stop-overs in recently rainfed areas in the Sahel [28, 30]. However, falcons from the Canary Islands displayed an even more pronounced zig-zag migration pattern than those from the westernmost Mediterranean colonies by migrating eastward for hundreds - thousands of km’s before reorienting southeast to Madagascar (Fig. S8). The latitude at which these longitudinal movements occurred was correlated with the ITF’s position (which varies depending on date, longitude and year), and we argue that flying near the ITF allows falcons to compensate for the drift over the desert in weaker winds than they could by travelling over more vegetated areas further south, where we might expect higher food availability (i.e. insects). However, it is also possible that the monsoon rain front south of the ITF is characterized by the presence of particularly profitable prey such as desert locusts [24]. By detouring far east across the Sahel falcons also seemed to reduce the distance flown over the equatorial rainforest by 500–1000 km compared to the GCR. Combined with a regional peak in nocturnal flight activity, this suggests that falcons perceive the tropical rainforest belt as a considerable barrier [28, 60]. Besides food availability, the humid tropical forests may constitute a migration barrier due to climatic conditions that hinder soaring (e.g. limited thermal convection) as well as flapping flight (e.g. precipitation). Falcons again reduced daily travel times and made irregular short stop-overs when reaching the East African savannahs. Importantly, they reduced headwind resistance through strong adverse wind fields here, showing how wind regimes themselves can form migration barriers even over hospitable landscapes [10].

In spring, the protracted trans-oceanic flight from northern Madagascar to East Africa was not associated with gains in wind support because this route happened to be closely aligned with the GCR to the Canary Islands. This behaviour should nevertheless be considered as an adaptive drift response because falcons from eastern colonies -for whom this route does represent a detour- also use this route to exploit strong prevailing winds [29, 31] (Fig. S8). Moreover, the highly mixed pattern of overdrift and overcompensation behaviour during spring migration indicates that falcons intended to reach some intermediate goals instead of leveraging wind support along the GCR directly. The majority of the Canarian falcons detoured into the Horn of Africa after reaching mainland Africa, like conspecifics from Mediterranean colonies (Fig. S8), confirming that this is a key stop-over region for the species as a whole, as it is for several other insectivorous Afro-Palearctic migrants in spring [61, 62]. Falcons are assumed to prey on large or superabundant insects following early spring rains in this region (so-called Belg rains [27, 63]). However, there is insufficient field data to determine what foraging opportunities explain the importance of this stop-over in the extant migratory network of our fringe study population. It has been suggested the stop-over is a genetically conserved strategy [27]. But while intense Belg rains have been a stable climatic feature in the Horn of Africa for millennia they have been steadily declining over the last century as a result of global warming [64]. Moreover, spring rains are subject to great inter-annual and sub-seasonal variability under current climatic conditions [65, 66] and droughts in the Horn of Africa have been linked to delayed spring migration and even failed reproduction of migrant birds [62]. In our study two-three falcons consistently forewent the Ethiopian stop-overs, stopping-over in rainfed areas in Uganda and South Sudan instead (Fig. S7). This indicates at least some flexibility in the development of spring routes, even in areas that seem to be of great significance for the species as a whole.

Frequent nocturnal travel over the eastern Sahel in spring suggests that the decision to travel eastward along the ITF is not motivated by local foraging opportunities, and rather by barrier avoidance. Taken together with previous tracking studies from Mediterranean colonies we suggest the existence of a migratory divide in spring desert-crossing corridors between western and eastern colonies (Fig. S8). The latter (e.g. Greek and Cypriotic falcons) tend to travel directly northward across the eastern Sahara or along the eastern Red Sea coast and reorient to the breeding colony after reaching the eastern Mediterranean region. By contrast, falcons from the Canary Islands and western Mediterranean colonies tend to first travel westward over the Sahel, thus postponing the desert-crossing to the west of 15E (Fig. S8). For Canarian Eleonora’s falcons, postponing the desert-crossing until Central or West Africa lengthens the spring migration by > 500 km but reduces the desert-crossing distance by > 1500 km as opposed to returning from Ethiopia along the GCR. Circumventing the Sahara along the eastern side would extend the spring migration by > 1000 km, with no obvious gains in wind support or foraging opportunities.

Even though previous studies reported low individual repeatability of seasonal route choice in Eleonora’s falcons, a common pattern in Africa-Eurasian migrants, we found evidence for substantial individual differences in spring stop-overs and the latitude at which falcons migrated across the Sahel-Sudan zone during spring migration, in contrast to falcons converging in a relatively narrow latitudinal corridor south of the ITF in autumn. In fact, trans-Sahelian routes were much more individually variable in spring, with individuals at one end of the spectrum overshooting the ITF over the eastern Sahel and initiating desert-crossings from Central Africa, while others wandered extensively in West Africa before crossing the desert. Individually repeatable spring stop-over sites, particularly in East Africa, seem to act as anchoring points for individual routes across years (Fig. S7). This finding, combined with the later, slower migration of juveniles along markedly different routes than adults [27, 30], suggests that route development is mediated by exploration-refinement learning [67–70]. Additional tracking studies involving juvenile birds would be needed to determine how orientation behaviour and migratory performance are refined through individual experience, and how they respond to intra-generational changes in environmental conditions.

## Conclusions

By contextualizing route choice and migratory performance patterns in prevailing winds and seasonal climates across distinct biomes we revealed a complex interplay of adaptive drift and barrier-avoidance responses in the trans-equatorial migration of a fly-forage migrant. Eleonora’s falcons engaged in adaptive drift to maximize wind support over ‘hard’ barriers (i.e. desert and sea) but, importantly, also through adverse wind fields over hospitable landscapes. By contrast, in weak or favourable wind fields falcons often leveraged wind support by detouring from the GCR, for example to exploit habitual spring stop-overs in the Horn of Africa and to circumvent the ‘soft’ barrier of the Congo Basin in autumn. Daily travel distances vary greatly depending on daily travel time budgets, with particularly long flights occurring over barriers. However, the favourable orientation of spring wind regimes for westward migration is what permitted falcons to travel faster when heading to their breeding grounds, rather than time-minimizing behaviours per se.

Longitudinal movements along the ITF were associated with reduced wind support in both seasons and were more individually variable in spring. Even though this study offers an extreme example of longitudinal migration, variation in trans-Saharan migration patterns among and within other species can likely be explained in part from common responses to the seasonally shifting position of the ITF and its associated atmospheric circulation patterns. For example, birds that cross the Sahara in August–September can be expected to escape the influence of strong desert winds and to encounter more hospitable conditions and calmer winds hundreds of km’s further north than those that cross in October–November. The ITF may thus offer a temporally dynamic coordinate system within which to compare migration patterns across populations and species.

## Supplementary Information

**Additional file 1 **: **Fig. S1**. (a) Flyway patterns in nocturnal flight activity and (b) daily flight activity-budgets of Eleonora’s falcons. **Fig. S2** (a) Biome-annotation of autumn and spring migration routes and (b) daily flight activity-budgets of Eleonora’s falcons across each biome in each season. **Fig. S3** Linear relationships of mean individual (a) trip duration, (b) stop-over days, (c) travel days, and (d) daily tailwind assistance with mean individual detour extent in autumn (red, *n* = 19) and spring (blue, *n* = 18). **Fig. S4** Regional differences in orientation behaviour in response to sidewinds towards the seasonal destination. **Fig. S5** Regional variation in (a) daily travel time, (b) daily beeline distances and (c) daily mean travel speeds during autumn (left) and spring (right) migration. **Fig. S6** Biome differences in performance and wind support metrics during autumn (left) and spring (right) migration. **Fig. S7** Individual differences in route choice. **Fig. S8** Approximate seasonal migration routes by adult Eleonora’s falcons from and to colonies across the breeding range.

**Additional file 2 **: **Table S1.** Seasonal summary statistics for 19 falcons tracked over a total of 75 migrations. **Table S2.** Summary statistics for seasonal performance per sex. **Table S3.** Selecting best GLMMs for trip-scale movement statistics (*n* = 75) as a function of detour extent and season. **Table S4.** Fixed effects of detour extent and season trip-scale movement statistics according to most parsimonious GLMMs (Table S2). **Table S5.** Selecting best GLMMs for daily movement statistics (*n* = 1842 travel days) as a function of tailwind along the track, biome and season. **Table S6.** Selecting best GLMMs for daily movement statistics (*n* = 1842 travel days) as a function of tailwind relative to the goal, biome and season. **Table S7.** Selecting the best GLMM for log-transformed daily mean travel speed (*n* = 1842 travel days).

**Additional file 3. **Codes for reproducing seasonal NDVI maps in Google Earth Engine. 

## Data Availability

Tracking data are stored in the UvA-BiTS database and available upon request. Annotated R code to reproduce the analyses is available via WMGV’s Github (https://github.com/Wouter-Vansteelant/Vansteelant-etal-2021-Movement-Ecology) and automatically installs all required packages. ITF positional data were downloaded from NOAA-CPC via http://www.cpc.ncep.noaa.gov/products/fews/ITCZ/itcz.shtml. All other weather data are freely available through NOAA and are automatically downloaded and processed by the R code we provided. Code to reproduce seasonal NDVI maps in Google Earth Engine is provided in Additional file 3.
