# Supplementary material for: Adaptive drift and barrier-avoidance by a fly-forage migrant along a climate-driven flyway
Source: Mov Ecol. 2021 Jul 13;9:37. doi: 10.1186/s40462-021-00272-8 (PMC8276455; doi:10.1186/s40462-021-00272-8)
Supplement: Supplementary file 1 — Additional file 1 : Fig. S1. (a) Flyway patterns in nocturnal flight activity and (b) daily flight activity-budgets of Eleonora’s falcons. Fig. S2 (a) Biome-annotation of autumn and spring migration routes and (b) daily flight activity-budgets of Eleonora’s falcons across each biome in each season. Fig. S3 Linear relationships of mean individual (a) trip duration, (b) stop-over days, (c) travel days, and (d) daily tailwind assistance with mean individual detour extent in autumn (red, n = 19) and spring (blue, n = 18). Fig. S4 Regional differences in orientation behaviour in response to sidewinds towards the seasonal destination. Fig. S5 Regional variation in (a) daily travel time, (b) daily beeline distances and (c) daily mean travel speeds during autumn (left) and spring (right) migration. Fig. S6 Biome differences in performance and wind support metrics during autumn (left) and spring (right) migration. Fig. S7 Individual differences in route choice. Fig. S8 Approximate seasonal migration routes by adult Eleonora’s falcons from and to colonies across the breeding range. [file 40462_2021_272_MOESM1_ESM.docx]

**
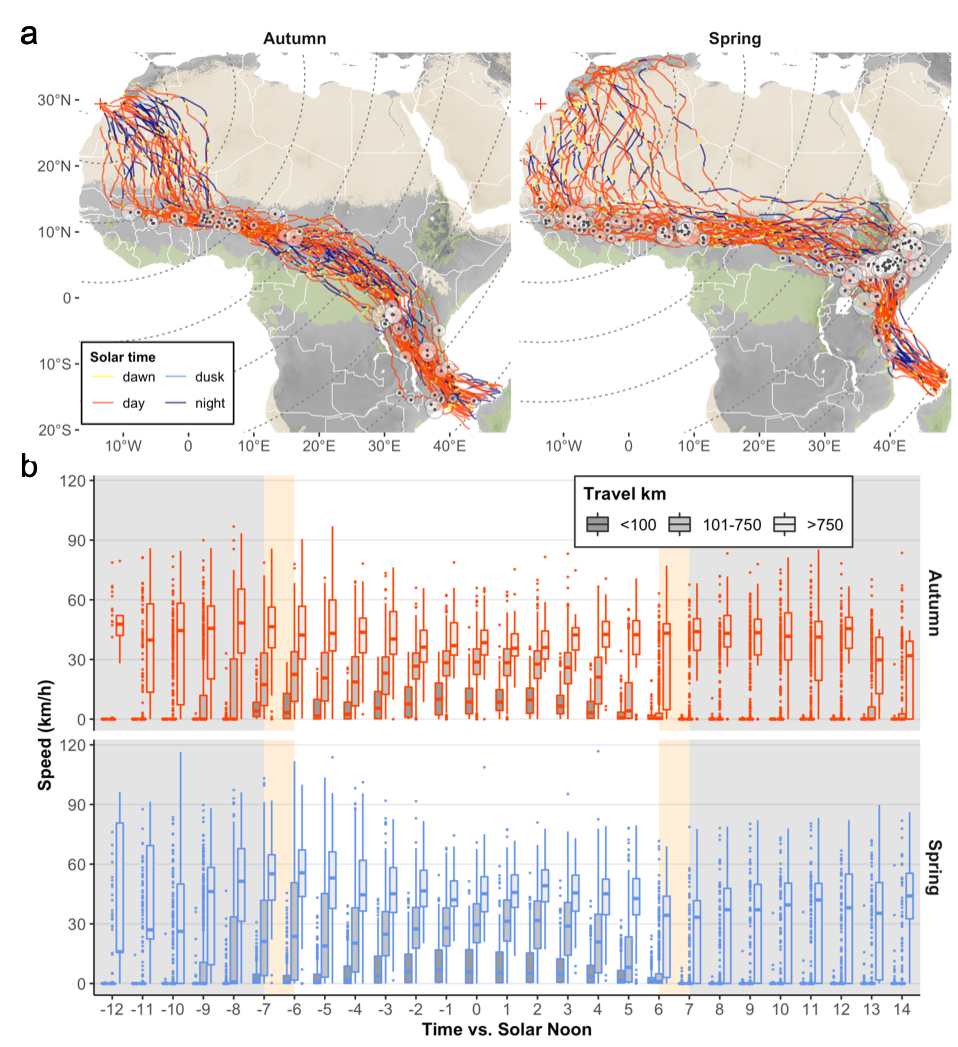
**

**Fig.S1 (a) Flyway patterns in nocturnal flight activity and (b) daily flight activity-budgets of Eleonora’s falcons.** (a) Nocturnal travel often occurred over the Sahara and the Indian Ocean in both seasons. Falcons also engaged in nocturnal travel over the tropical rainforest belt in autumn, and across the eastern Sahel-Sudan zone in spring (Fig.S3a). Dashed grey lines indicate distance to the colony at 1000km intervals. (b) Daily time budgets on stop-over days (<100 km d^-1^), travel days (>100 km d^-1^) and long-distance travel days (>750 km d^-1^) in autumn (red) and spring (blue). Boxplots show the median (thick line), the inter-quartile range (IQR, boxes), the 1.5*IQR range (whiskers) and outliers (points). Shaded areas correspond to night-time (grey), daytime (white), and dawn/dusk (orange). The median and IQR of hourly speeds on stop-over days fall (well) below 25km h^-1^ throughout the daylight period. By contrast, on days classified as travel days falcons typically achieve speeds between 25-50 km h^-1^ throughout most of the daylight period, and often engaging in active travel from 2 hours prior to sunrise, and on most occasions during mid-day. Long-distance travel days (>750 km d^-1^) were characterized by relatively fast hourly speeds during daytime travel, and frequent nocturnal travel.


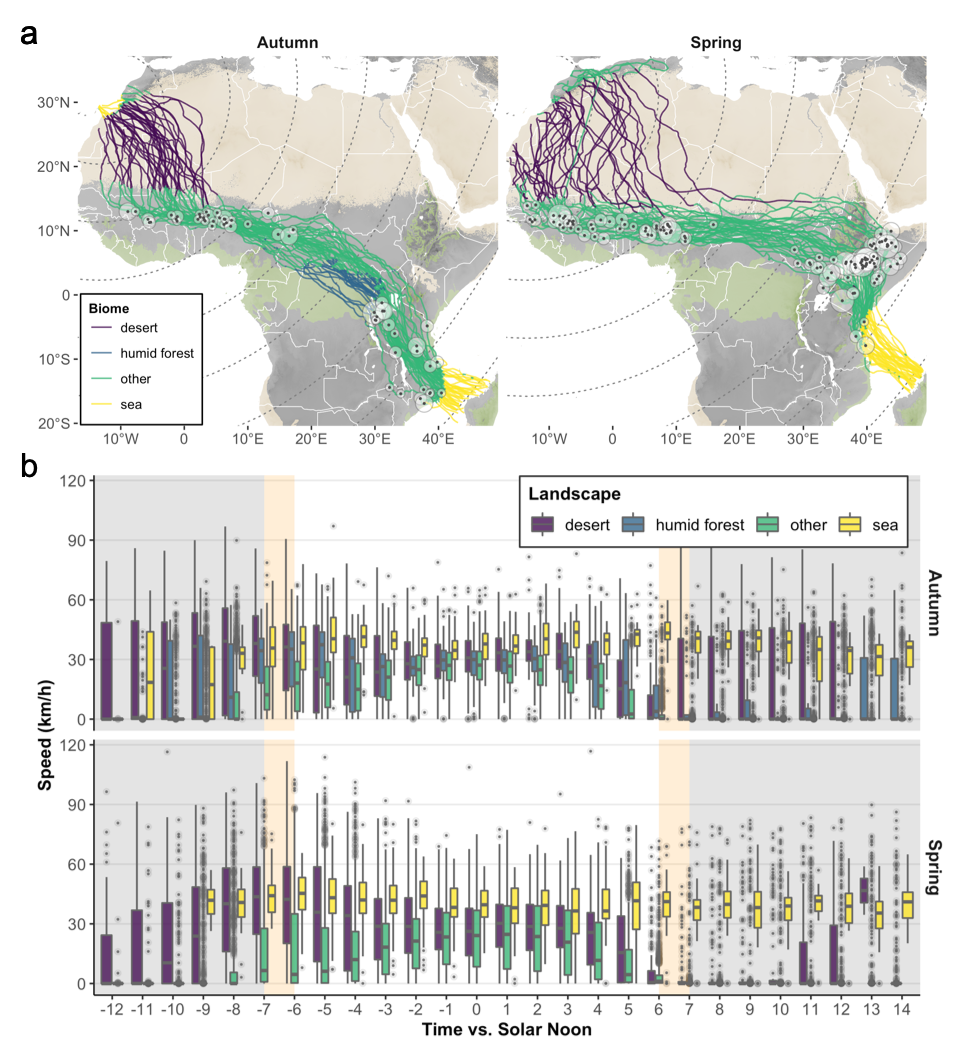


**Fig.S2 (a) Biome-annotation of autumn and spring migration routes and (b) daily flight activity-budgets of Eleonora’s falcons across each biome in each season.** (a) In order to focus on major barrier-crossing events (Sahara, Congo Basin rainforest, Indian Ocean) only the longest continuous flight over desert biome was annotated for each season, ‘humid forest’ biome outside the Congo Basin was ignored for annotating forest-crossings, and fixes over islands were included in sea-crossing segments. (b) We show hourly travel speeds vs. solar time of day in each biome during autumn (top) and spring (bottom). Over the sea (yellow) falcons necessarily continue flying at night. Falcons also clearly show extended and faster morning and evening activity over the desert, and to lesser extent over the tropical forest, compared to other -more hospitable- biomes.


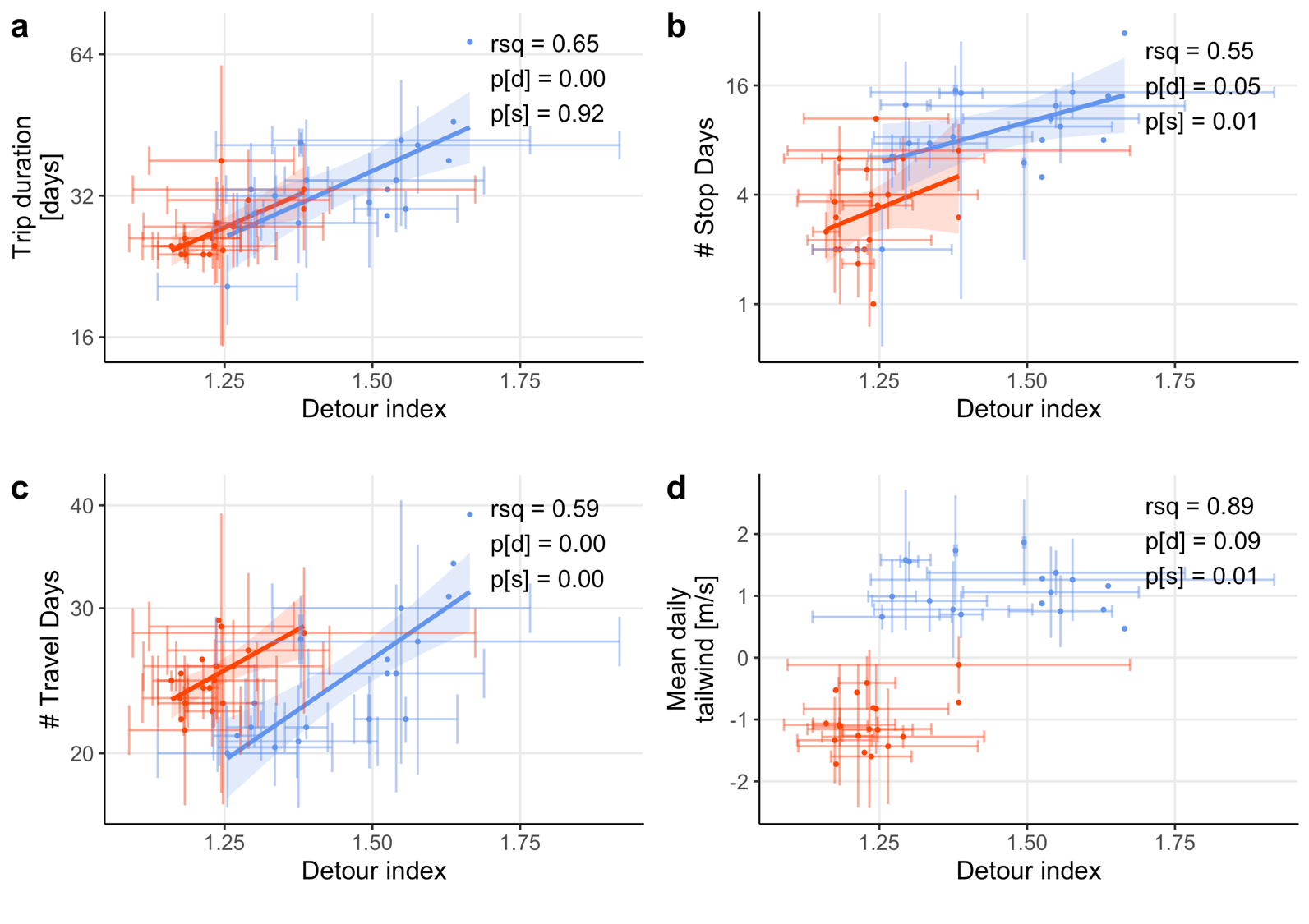


**Fig.S3 Linear relationships of mean individual (a) trip duration, (b) stop-over days, (c) travel days, and (d) daily tailwind assistance with mean individual detour extent in autumn (red, n = 19) and spring (blue, n = 18).** Points and crossbars show the mean (±se) values per season and individual. Regression lines (±CI) are only shown if detour extent had a significant effect according to a linear regression model formulated as y ~ detour + season, whereby the response variables mean individual trip duration, stop-over days and travel days were log-transformed. Labels show R^2^ (rsq) and p-values for the effects of detour (p[d]) and season (p[s]).


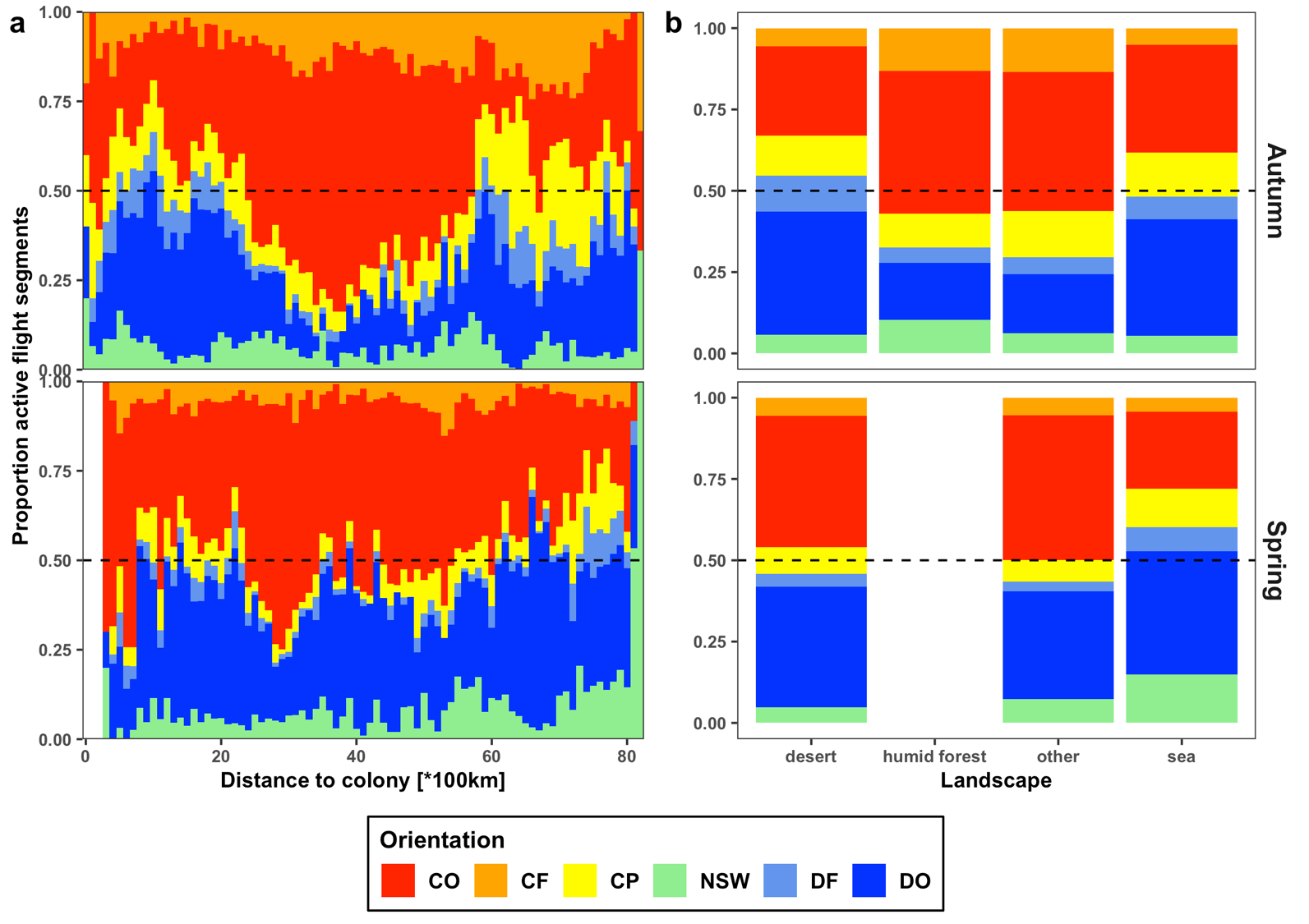


**Fig.S4 Regional differences in orientation behaviour in response to sidewinds towards the seasonal destination.** (a) There are clear peaks in partial compensation and particularly (over)drift behaviour in the first and second third of the autumn migration. By contrast, falcons mostly overcompensated for sidewinds during the middle section of the autumn migration. In spring, the proportion of overcompensation and over-drift behaviour shows less pronounced regional patterns, with an overall higher occurrence of overdrift than in autumn. Slight peaks in overcompensation behaviour occurred just before and after the desert crossing. (b) The proportion of each orientation behaviour summarized per biome and season. Note the high propensity for (over)drift over desert and sea in autumn, and the generally higher frequency of overdrift during spring compared to autumn.

**Fig.S5 Regional variation in (a) daily travel time, (b) daily beeline distances and (c) daily mean travel speeds during autumn (left) and spring (right) migration.** Dashed grey lines indicate distance to the colony at 1000km intervals.


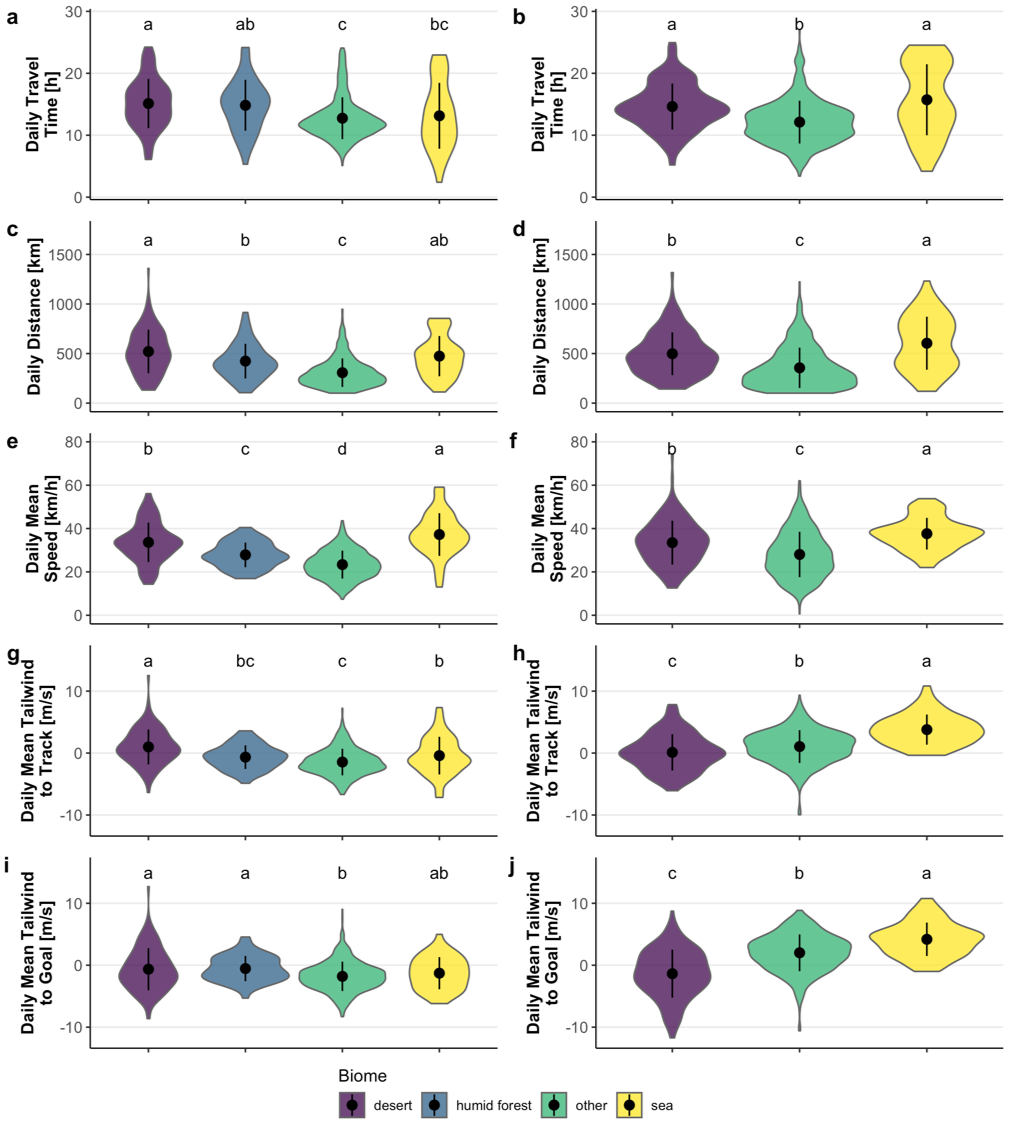


**Fig.S6 Biome differences in performance and wind support metrics during autumn (left) and spring(right) migration.** Labels indicate significant differences between biomes according to a Tukey post-hoc comparison between groups.

**
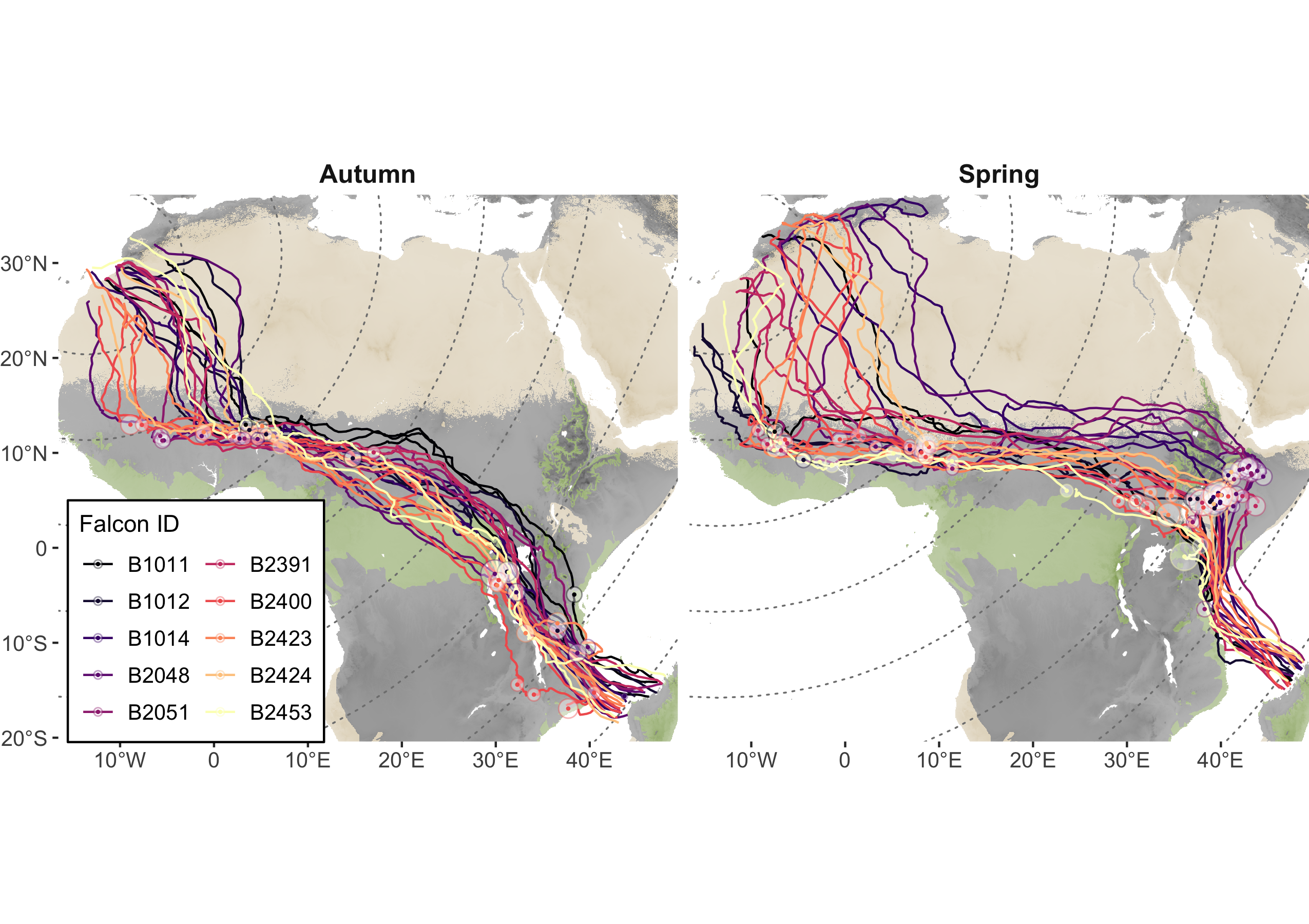
**

**Fig.S7 Individual differences in route choice.** To ensure legibility of the maps we show a subset of 10 individuals (colour legend) for which at least two full migration cycles were recorded. Migration corridors show substantial overlap between individuals and converge across the western Sahel and near the Mozambique Channel in autumn, and to a lesser extent across the Sahel in spring. Nevertheless, some individuals (e.g. B2453 and B2424) have markedly narrow migration corridors within the seasonal flyways, and display marked differences in route choice with other individuals during at least some stages (such as their autumn routes across the Congo Basin). We also note a general tendency of falcons to use individually consistent stop-over areas within the East African stop-over region, whereby some stopped-over relatively far north in Ethiopia and Somalia and then tended to return via more northerly routes across the eastern Sahel (north of the ITF, see Fig.2) initiating earlier desert-crossings (thereby foregoing stop-overs in West Africa and reducing total trip duration) (e.g. B1014, B2048). Nevertheless, we also found evidence of highly flexible route choice, especially during sea and desert-crossings. For example, the spring desert-crossings of B1011 and B1014 differed by >1000km longitude between years.

**
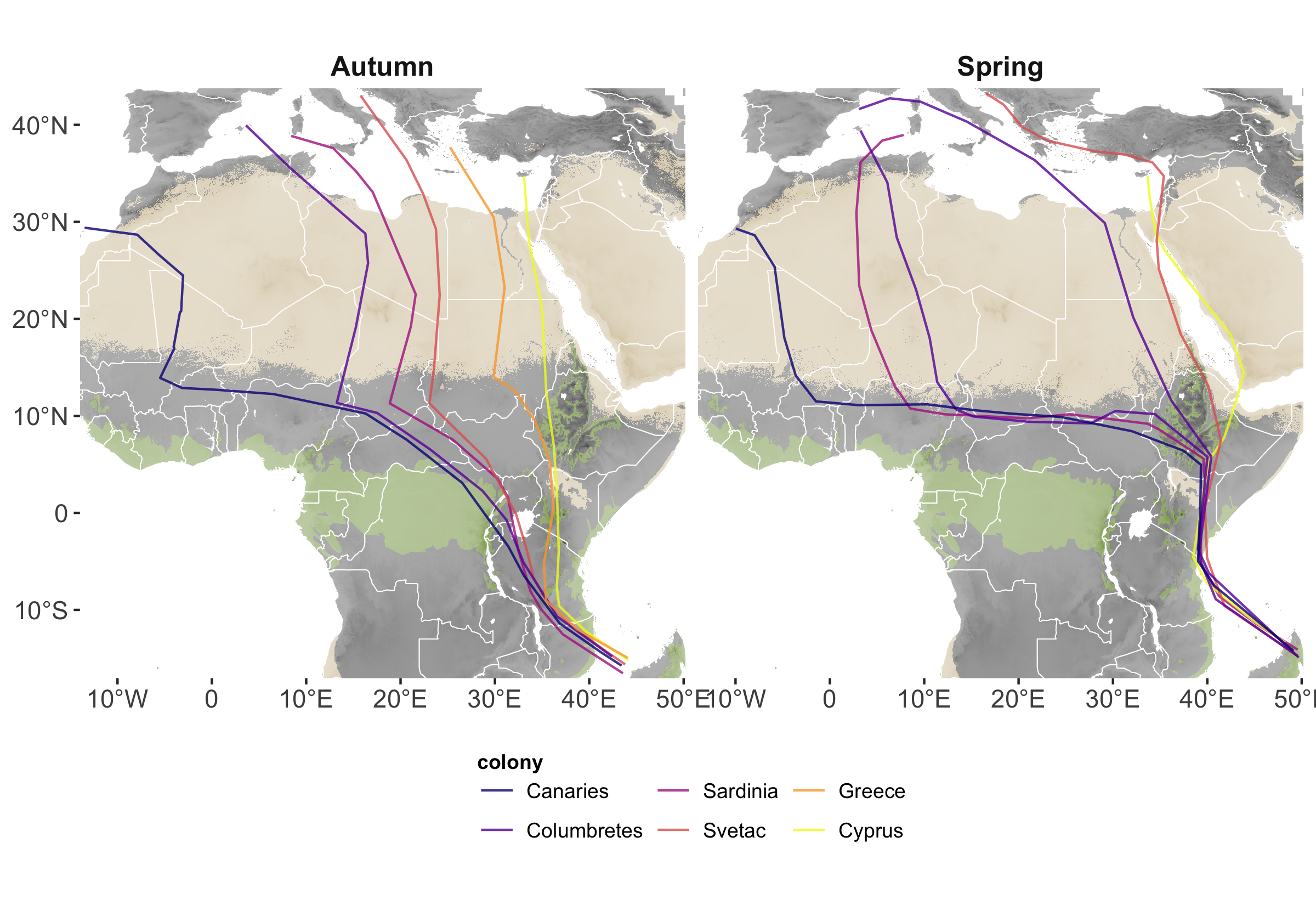
**

**Fig.S8 Approximate seasonal migration routes by adult Eleonora’s falcons from and to colonies across the breeding range.** Mean seasonal routes were manually drawn in Google Earth based on previously published tracking data from Columbretes/Balearic Isles [28, 30], Sardinia [27], Svetac (Croatia) [30], Greece [29] and Cyprus [31], as well as for our Canarian study population. For Greece no spring data has been published. Note that for falcons breeding on the Columbretes/Balearic Isles we draw two spring migration routes to reflect the fact that some individuals cross the eastern Sahara before reorienting westward along the northern Mediterranean coast, whereas others migrate westward south of the Sahara before crossing the desert (cf. individuals from Sardinia and Canary Islands).
