# Supplementary material for: Adaptive drift and barrier-avoidance by a fly-forage migrant along a climate-driven flyway
Source: Mov Ecol. 2021 Jul 13;9:37. doi: 10.1186/s40462-021-00272-8 (PMC8276455; doi:10.1186/s40462-021-00272-8)
Supplement: Supplementary file 2 — Additional file 2 : Table S1. Seasonal summary statistics for 19 falcons tracked over a total of 75 migrations. Table S2. Summary statistics for seasonal performance per sex. Table S3. Selecting best GLMMs for trip-scale movement statistics (n = 75) as a function of detour extent and season. Table S4. Fixed effects of detour extent and season trip-scale movement statistics according to most parsimonious GLMMs (Table S2). Table S5. Selecting best GLMMs for daily movement statistics (n = 1842 travel days) as a function of tailwind along the track, biome and season. Table S6. Selecting best GLMMs for daily movement statistics (n = 1842 travel days) as a function of tailwind relative to the goal, biome and season. Table S7. Selecting the best GLMM for log-transformed daily mean travel speed (n = 1842 travel days). [file 40462_2021_272_MOESM2_ESM.docx]

**Table S1. Seasonal summary statistics for 19 falcons tracked over a total of 75 migrations.**

| **Season** | **Sex** | **ID** | **n** | **Cumulative Dist [km]** | | **Detour index** | **Start date** | | **End date** | | **Duration [days]** | | **Stop-over days** | | **Travel days** | |
| --- | --- | --- | --- | --- | --- | --- | --- | --- | --- | --- | --- | --- | --- | --- | --- | --- |
|  |  |  |  | **mean** | **SD** |  | **mean** | **SD** | **mean** | **SD** | **mean** | **SD** | **mean** | **SD** | **mean** | **SD** |
| **Autumn** | **Female** | B1011 | 3 | 9087 | 392 | 1.2 | Oct-14 | 3 | Nov-06 | 3 | 24 | 1 | 1 | 1 | 23 | 1 |
|  |  | B1014 | 4 | 9474 | 838 | 1.2 | Oct-21 | 5 | Nov-14 | 5 | 25 | 4 | 1 | 2 | 25 | 3 |
|  |  | B2380 | 1 | 9128 | - | 1.2 | Oct-18 | - | Nov-10 | - | 24 | - | 2 | - | 22 | - |
|  |  | B2394 | 1 | 9672 | - | 1.2 | Oct-17 | - | Nov-10 | - | 24 | - | 1 | - | 24 | - |
|  |  | B2400 | 3 | 9852 | 975 | 1.3 | Oct-15 | 2 | Nov-15 | 8 | 31 | 9 | 5 | 2 | 27 | 6 |
|  |  | B2423 | 2 | 10620 | 1746 | 1.4 | Oct-21 | 1 | Nov-22 | 8 | 33 | 8 | 6 | 3 | 28 | 6 |
|  |  | B2424 | 2 | 8948 | 267 | 1.2 | Oct-25 | 4 | Nov-19 | 4 | 25 | 0 | 2 | 1 | 25 | 1 |
|  |  | B2447 | 1 | 9498 | - | 1.2 | Oct-15 | - | Nov-09 | - | 25 | - | 1 | - | 25 | - |
|  |  | B2450 | 1 | 9476 | - | 1.2 | Oct-19 | - | Nov-14 | - | 26 | - | 1 | - | 26 | - |
|  | **Male** | B1012 | 3 | 9107 | 753 | 1.2 | Oct-22 | 4 | Nov-16 | 3 | 26 | 2 | 5 | 3 | 21 | 4 |
|  |  | B1013 | 2 | 9602 | 460 | 1.2 | Oct-19 | 5 | Nov-13 | 4 | 26 | 1 | 5 | 1 | 23 | 2 |
|  |  | B2048 | 2 | 9690 | 1124 | 1.2 | Oct-20 | 9 | Nov-26 | 13 | 38 | 23 | 10 | 12 | 29 | 11 |
|  |  | B2051 | 2 | 9599 | 378 | 1.2 | Oct-19 | 8 | Nov-15 | 1 | 28 | 7 | 3 | 3 | 26 | 4 |
|  |  | B2337 | 1 | 10854 | - | 1.4 | Oct-23 | - | Nov-25 | - | 30 | - | 2 | - | 28 | - |
|  |  | B2368 | 2 | 9914 | 975 | 1.3 | Oct-25 | 4 | Nov-21 | 1 | 28 | 5 | 3 | 1 | 26 | 4 |
|  |  | B2378 | 1 | 9544 | - | 1.2 | Oct-18 | - | Nov-15 | - | 28 | - | 0 | - | 29 | - |
|  |  | B2391 | 3 | 9456 | 119 | 1.2 | Oct-25 | 4 | Nov-17 | 6 | 24 | 2 | 1 | 1 | 24 | 2 |
|  |  | B2392 | 3 | 9283 | 397 | 1.2 | Oct-19 | 4 | Nov-12 | 3 | 25 | 1 | 3 | 3 | 23 | 2 |
|  |  | B2453 | 2 | 9917 | 489 | 1.2 | Oct-20 | 8 | Nov-13 | 1 | 25 | 9 | 3 | 4 | 23 | 6 |
| **Spring** | **Female** | B1011 | 2 | 9880 | 833 | 1.3 | Apr-09 | 5 | Apr-29 | 1 | 21 | 4 | 1 | 1 | 20 | 3 |
|  |  | B1014 | 3 | 10536 | 1060 | 1.4 | Apr-12 | 3 | May-09 | 6 | 28 | 5 | 7 | 2 | 21 | 4 |
|  |  | B2380 | 1 | 11646 | - | 1.5 | Apr-11 | - | May-10 | - | 29 | - | 4 | - | 25 | - |
|  |  | B2394 | 1 | 11829 | - | 1.5 | Apr-05 | - | May-07 | - | 33 | - | 7 | - | 26 | - |
|  |  | B2400 | 3 | 12209 | 2493 | 1.6 | Apr-04 | 4 | May-14 | 13 | 41 | 9 | 14 | 4 | 27 | 9 |
|  |  | B2423 | 2 | 11491 | 118 | 1.5 | Apr-12 | 6 | May-23 | 1 | 31 | 8 | 5 | 4 | 22 | 3 |
|  |  | B2424 | 2 | 10511 | 33 | 1.4 | Apr-10 | 6 | May-31 | 4 | 42 | 2 | 14 | 6 | 28 | 4 |
|  |  | B2447 | 1 | 12910 | - | 1.7 | Apr-02 | - | Jun-01 | - | 68 | - | 30 | - | 39 | - |
|  |  | B2450 | 1 | 12483 | - | 1.6 | Apr-02 | - | May-28 | - | 46 | - | 13 | - | 34 | - |
|  | **Male** | B1012 | 3 | 10349 | 142 | 1.3 | Apr-13 | 7 | May-11 | 2 | 29 | 6 | 7 | 3 | 23 | 3 |
|  |  | B1013 | 2 | 10215 | 264 | 1.3 | Apr-10 | 0 | May-07 | 5 | 28 | 5 | 6 | 2 | 21 | 0 |
|  |  | B2048 | 2 | 11669 | 240 | 1.6 | Apr-11 | 13 | May-20 | 11 | 30 | 3 | 9 | 4 | 22 | 1 |
|  |  | B2051 | 2 | 11858 | 1194 | 1.5 | Apr-01 | 1 | May-04 | 6 | 35 | 8 | 10 | 1 | 25 | 7 |
|  |  | B2368 | 2 | 10845 | 319 | 1.4 | Apr-07 | 1 | May-11 | 13 | 35 | 12 | 14 | 13 | 22 | 1 |
|  |  | B2378 | 1 | 12703 | - | 1.6 | Apr-01 | - | May-09 | - | 38 | - | 7 | - | 31 | - |
|  |  | B2391 | 3 | 10523 | 716 | 1.3 | Apr-08 | 5 | May-09 | 13 | 32 | 9 | 7 | 2 | 20 | 2 |
|  |  | B2392 | 3 | 12142 | 1694 | 1.5 | Apr-07 | 7 | May-18 | 18 | 42 | 14 | 11 | 3 | 30 | 11 |
|  |  | B2453 | 2 | 10179 | 258 | 1.3 | Apr-06 | 9 | May-18 | 1 | 33 | 8 | 12 | 9 | 22 | 1 |

n indicates the number of trips recorded per season per bird**.**

**Table S2. Summary statistics for seasonal performance per sex.**

| **Season** | **Sex** | **n** | **Cumulative Dist [km]** | | **Detour index** | | **Duration [days]** | | **Stop-over days** | | **Travel days** | |
| --- | --- | --- | --- | --- | --- | --- | --- | --- | --- | --- | --- | --- |
|  |  |  | **mean** | **SD** | **mean** | **SD** | **mean** | **SD** | **mean** | **SD** | **mean** | **SD** |
| Autumn | Female | 18 | 9528 | 502 | 1.23 | 0.07 | 26 | 3 | 2 | 2 | 25 | 2 |
|  | Male | 21 | 9697 | 478 | 1.24 | 0.06 | 28 | 4 | 3 | 3 | 25 | 3 |
| Spring | Female | 16 | 11500 | 1007 | 1.49 | 0.13 | 38 | 14 | 11 | 9 | 27 | 6 |
|  | Male | 20 | 11165 | 943 | 1.43 | 0.14 | 33 | 5 | 9 | 3 | 24 | 4 |

n indicates the number of trips recorded per season per sex**.**

**Table S3. Selecting best GLMMs for trip-scale movement statistics (n = 75) as a function of detour extent and season.**

| **Response. variable** | **Model** | **df** | **AICc** | **∆AICc** | **AICc Weight** | **R2 model** | **R2 fixed** | **R2 random** |
| --- | --- | --- | --- | --- | --- | --- | --- | --- |
| **Log(Season Duration)** | detour | 4 | -37.29 | 0.00 | 0.66 | 0.54 | 0.54 | 0.00 |
|  | detour+season | 5 | -35.11 | 2.18 | 0.22 | 0.54 | 0.54 | 0.00 |
|  | detour*season | 6 | -33.81 | 3.49 | 0.12 | 0.54 | 0.54 | 0.00 |
|  | season | 4 | 5.05 | 42.34 | 0.00 | 0.22 | 0.18 | 0.04 |
|  |  | 3 | 17.98 | 55.28 | 0.00 | 0.02 | 0.00 | 0.02 |
| **Log(Stop days+1)** | detour+season | 6 | 155.88 | 0.00 | 0.62 | 0.56 | 0.44 | 0.12 |
|  | detour*season | 7 | 157.05 | 1.16 | 0.35 | 0.57 | 0.45 | 0.12 |
|  | season | 5 | 162.96 | 7.07 | 0.02 | 0.50 | 0.37 | 0.14 |
|  | detour | 5 | 164.00 | 8.12 | 0.01 | 0.48 | 0.34 | 0.14 |
|  |  | 4 | 194.79 | 38.91 | 0.00 | 0.19 | 0.00 | 0.19 |
| **Log(Travel days)** | detour+season | 6 | -100.07 | 0.00 | 0.74 | 0.62 | 0.62 | 0.00 |
|  | detour*season | 7 | -97.95 | 2.12 | 0.26 | 0.63 | 0.62 | 0.01 |
|  | detour | 5 | -61.74 | 38.33 | 0.00 | 0.36 | 0.34 | 0.02 |
|  |  | 4 | -33.22 | 66.84 | 0.00 | 0.04 | 0.00 | 0.04 |
|  | season | 5 | -31.19 | 68.88 | 0.00 | 0.04 | 0.00 | 0.04 |
| **Tailwind** | detour*season | 6 | 150.94 | 0.00 | 0.72 | 0.77 | 0.77 | 0.00 |
|  | season | 4 | 153.76 | 2.82 | 0.18 | 0.75 | 0.75 | 0.00 |
|  | detour+season | 5 | 154.75 | 3.81 | 0.11 | 0.75 | 0.75 | 0.00 |
|  | detour | 4 | 227.31 | 76.37 | 0.00 | 0.33 | 0.33 | 0.00 |
|  |  | 3 | 255.66 | 104.72 | 0.00 | 0.00 | 0.00 | 0.00 |

All models allow for randomly varying intercepts per individual and year. We select the best model for each response variable as the most parsimonious model for which ∆AICc < 2. These are also considered the most likely models based on AICc weights.

**Table S4. Fixed effects of detour extent and season trip-scale movement statistics according to most parsimonious GLMMs (Table S2).**

| **Response variable** | **Model coefficient** | **Estimate** | **Std. Error** | **t value** | **Pr(>\|t\|)** |
| --- | --- | --- | --- | --- | --- |
| **Log(Season Duration)** | Intercept | 1.879 | 0.163 | 11.547 | <0.001 |
|  | detour | 1.129 | 0.121 | 9.304 | <0.001 |
| **Log(Stop days +1)** | Intercept | -1.021 | 0.683 | -1.496 | 0.139 |
|  | detour | 1.749 | 0.547 | 3.197 | 0.002 |
|  | season | 0.626 | 0.183 | 3.424 | 0.001 |
| **Log(Travel days)** | Intercept | 1.875 | 0.122 | 15.377 | <0.001 |
|  | detour | 1.072 | 0.098 | 10.930 | <0.001 |
|  | season | -0.245 | 0.033 | -7.363 | <0.001 |
| **Log(Tailwind_to_ Track)** | Intercept | -4.546 | 1.248 | -3.644 | <0.001 |
|  | detour | 2.819 | 1.010 | 2.791 | 0.007 |
|  | season | 5.926 | 1.525 | 3.886 | <0.001 |
|  | detour:season | -2.989 | 1.178 | -2.537 | 0.013 |

We consider coefficient estimates to be significant if P<0.05 (bold).

**Table S5. Selecting best GLMMs for daily movement statistics (n = 1842 travel days) as a function of tailwind along the track, biome and season.**

| **Response variable** | **Model (fixed effects)** | **df** | **AIC** | **∆AIC** | **AICc Weight** | **R2 full** | **R2 fixed** | **R2 random** |
| --- | --- | --- | --- | --- | --- | --- | --- | --- |
| **Daily Travel Time** | biome | 7 | 12223 | 0 | 0.40 | 0.00 | 0.00 | 0.00 |
|  | season * biome | 10 | 12225 | 1 | 0.22 | 0.02 | 0.02 | 0.00 |
|  | season + biome | 8 | 12225 | 2 | 0.15 | 0.02 | 0.02 | 0.00 |
|  | tailwind_track + biome | 8 | 12225 | 2 | 0.15 | 0.02 | 0.02 | 0.00 |
|  | tailwind_track + season + biome | 9 | 12227 | 4 | 0.06 | 0.00 | 0.00 | 0.00 |
|  | tailwind_track * season * biome | 17 | 12229 | 6 | 0.02 | 0.03 | 0.03 | 0.00 |
|  | tailwind_track * biome | 11 | 12231 | 7 | 0.01 | 0.03 | 0.02 | 0.00 |
|  |  | 4 | 12253 | 30 | 0.00 | 0.00 | 0.00 | 0.00 |
|  | tailwind_track | 5 | 12254 | 30 | 0.00 | 0.02 | 0.02 | 0.00 |
|  | season | 5 | 12255 | 32 | 0.00 | 0.00 | 0.00 | 0.00 |
|  | tailwind_track + season | 6 | 12255 | 32 | 0.00 | 0.00 | 0.00 | 0.00 |
|  | tailwind_track * season | 7 | 12256 | 33 | 0.00 | 0.02 | 0.02 | 0.00 |
| **log(Daily Distance)** | ***tailwind_track + biome*** | ***8*** | ***2625*** | ***0*** | ***0.33*** | ***0.15*** | ***0.14*** | ***0.01*** |
|  | tailwind_track * biome | 11 | 2625 | 0 | 0.30 | 0.15 | 0.14 | 0.01 |
|  | tailwind_track * season * biome | 17 | 2626 | 1 | 0.24 | 0.19 | 0.18 | 0.01 |
|  | tailwind_track + season + biome | 9 | 2627 | 2 | 0.12 | 0.08 | 0.07 | 0.01 |
|  | season * biome | 10 | 2700 | 75 | 0.00 | 0.18 | 0.18 | 0.01 |
|  | season + biome | 8 | 2702 | 77 | 0.00 | 0.18 | 0.17 | 0.01 |
|  | biome | 7 | 2712 | 87 | 0.00 | 0.08 | 0.07 | 0.01 |
|  | tailwind_track | 5 | 2839 | 214 | 0.00 | 0.14 | 0.13 | 0.01 |
|  | tailwind_track + season | 6 | 2841 | 216 | 0.00 | 0.08 | 0.07 | 0.01 |
|  | tailwind_track * season | 7 | 2842 | 217 | 0.00 | 0.18 | 0.17 | 0.01 |
|  | season | 5 | 2957 | 332 | 0.00 | 0.02 | 0.01 | 0.01 |
|  |  | 4 | 2969 | 344 | 0.00 | 0.01 | 0.00 | 0.01 |
| **log(Daily Mean Speed)** | tailwind_track * season *biome | 17 | 517 | 0 | 0.99 | 0.29 | 0.29 | 0.01 |
|  | tailwind_track + season + biome | 9 | 527 | 9 | 0.01 | 0.19 | 0.18 | 0.01 |
|  | tailwind_track * biome | 11 | 550 | 33 | 0.00 | 0.22 | 0.22 | 0.01 |
|  | tailwind_track + biome | 8 | 553 | 36 | 0.00 | 0.21 | 0.21 | 0.01 |
|  | season * biome | 10 | 677 | 160 | 0.00 | 0.28 | 0.27 | 0.01 |
|  | season + biome | 8 | 702 | 184 | 0.00 | 0.28 | 0.28 | 0.01 |
|  | tailwind_track * season | 7 | 757 | 240 | 0.00 | 0.27 | 0.27 | 0.01 |
|  | tailwind_track + season | 6 | 764 | 247 | 0.00 | 0.18 | 0.16 | 0.01 |
|  | tailwind_track | 5 | 785 | 267 | 0.00 | 0.18 | 0.16 | 0.02 |
|  | biome | 7 | 803 | 285 | 0.00 | 0.18 | 0.17 | 0.01 |
|  | season | 5 | 1001 | 484 | 0.00 | 0.07 | 0.06 | 0.01 |
|  |  | 4 | 1101 | 584 | 0.00 | 0.04 | 0.00 | 0.04 |

All models allow for randomly varying intercepts between individuals and years. Daily distance and daily mean travel speed were log-transformed. We select the best model for each response variable as the most parsimonious model for which ∆AICc < 2. These are also considered the most likely models based on AICc weights. Note that the best model for daily travel time does not explain a high amount of variation and is therefore not considered to be a good model.

**Table S6. Selecting best GLMMs for daily movement statistics (n = 1842 travel days) as a function of tailwind relative to the goal, biome and season.**

| **Response variable** | **Model (fixed effects)** | **df** | **AIC** | **∆AIC** | **AIC Weights** | **R2 full** | **R2 fixed** | **R2 random** |
| --- | --- | --- | --- | --- | --- | --- | --- | --- |
| **Daily Travel Time** | tailwind_goal * season * biome | 17 | 12217 | 0 | 1 | 0.04 | 0.03 | 0.00 |
|  | biome | 7 | 12223 | 6 | 0 | 0.00 | 0.00 | 0.00 |
|  | season * biome | 10 | 12225 | 7 | 0 | 0.02 | 0.02 | 0.00 |
|  | tailwind_goal + biome | 8 | 12225 | 8 | 0 | 0.02 | 0.02 | 0.00 |
|  | season + biome | 8 | 12225 | 8 | 0 | 0.02 | 0.02 | 0.00 |
|  | tailwind_goal + season + biome | 9 | 12227 | 10 | 0 | 0.01 | 0.01 | 0.00 |
|  | tailwind_goal * biome | 11 | 12231 | 14 | 0 | 0.03 | 0.02 | 0.00 |
|  | tailwind_goal * season | 7 | 12246 | 29 | 0 | 0.02 | 0.02 | 0.00 |
|  |  | 4 | 12253 | 36 | 0 | 0.00 | 0.00 | 0.00 |
|  | tailwind_goal | 5 | 12255 | 38 | 0 | 0.02 | 0.02 | 0.00 |
|  | season | 5 | 12255 | 38 | 0 | 0.00 | 0.00 | 0.00 |
|  | tailwind_goal + season | 6 | 12257 | 40 | 0 | 0.00 | 0.00 | 0.00 |
| **log(Daily Distance)** | tailwind_goal * season * biome | 17 | 2668 | 0 | 1 | 0.17 | 0.16 | 0.01 |
|  | tailwind_goal + biome | 8 | 2678 | 11 | 0 | 0.15 | 0.14 | 0.01 |
|  | tailwind_goal + season + biome | 9 | 2680 | 12 | 0 | 0.04 | 0.03 | 0.01 |
|  | tailwind_goal * biome | 11 | 2682 | 15 | 0 | 0.15 | 0.14 | 0.01 |
|  | season * biome | 10 | 2700 | 33 | 0 | 0.16 | 0.15 | 0.01 |
|  | season + biome | 8 | 2702 | 35 | 0 | 0.16 | 0.15 | 0.01 |
|  | biome | 7 | 2712 | 44 | 0 | 0.02 | 0.02 | 0.01 |
|  | tailwind_goal * season | 7 | 2911 | 243 | 0 | 0.16 | 0.15 | 0.01 |
|  | tailwind_goal + season | 6 | 2942 | 274 | 0 | 0.02 | 0.02 | 0.01 |
|  | tailwind_goal | 5 | 2942 | 275 | 0 | 0.14 | 0.13 | 0.01 |
|  | season | 5 | 2957 | 290 | 0 | 0.02 | 0.01 | 0.01 |
|  |  | 4 | 2969 | 302 | 0 | 0.01 | 0.00 | 0.01 |
| **log(Daily Mean Speed)** | tailwind_goal * season * biome | 17 | 624 | 0 | 1 | 0.25 | 0.25 | 0.01 |
|  | tailwind_goal + season + biome | 9 | 636 | 13 | 0 | 0.11 | 0.10 | 0.01 |
|  | tailwind_goal * biome | 11 | 665 | 41 | 0 | 0.22 | 0.22 | 0.01 |
|  | tailwind_goal + biome | 8 | 666 | 43 | 0 | 0.21 | 0.21 | 0.01 |
|  | season * biome | 10 | 677 | 54 | 0 | 0.23 | 0.22 | 0.01 |
|  | season + biome | 8 | 702 | 78 | 0 | 0.24 | 0.23 | 0.01 |
|  | biome | 7 | 803 | 179 | 0 | 0.10 | 0.09 | 0.01 |
|  | tailwind_goal * season | 7 | 921 | 297 | 0 | 0.23 | 0.22 | 0.01 |
|  | tailwind_goal + season | 6 | 947 | 324 | 0 | 0.08 | 0.07 | 0.01 |
|  | tailwind_goal | 5 | 985 | 361 | 0 | 0.18 | 0.16 | 0.02 |
|  | season | 5 | 1001 | 378 | 0 | 0.07 | 0.06 | 0.01 |
|  |  | 4 | 1101 | 478 | 0 | 0.04 | 0.00 | 0.04 |

All models allow for random variation of intercepts between individuals and years. Daily distance and daily mean travel speed were log-transformed. We select the best model for each response variable as the most parsimonious model for which ∆AICc < 2. These are also considered the most likely models based on AICc weights. Note that the best model for daily travel time does not explain a high amount of variation and is therefore not considered to be a good model.

**Table S7. Selecting the best GLMM for log-transformed daily mean travel speed (n = 1842 travel days).**

| **Model (fixed effects)** | **df** | **AIC** | **∆AIC** | **AIC weight** | **R2 full** | **R2 fixed** | **R2 random** |
| --- | --- | --- | --- | --- | --- | --- | --- |
| ~ (tailwind_track + abs(sidewind_track)) * travel_hrs * biome | 27 | 87.33 | 0.00 | 1.00 | 0.45 | 0.43 | 0.01 |
| ~ (tailwind_track + abs(sidewind_track)) * biome + season_spring | 12 | 145.32 | 57.99 | 0.00 | 0.29 | 0.28 | 0.01 |
| ~ biome * travel_hrs | 9 | 151.68 | 64.35 | 0.00 | 0.36 | 0.33 | 0.04 |
| ~ (tailwind_track + abs(sidewind_track)) * travel_hrs + biome | 15 | 210.76 | 123.43 | 0.00 | 0.42 | 0.41 | 0.01 |
| ~ travel_hrs * abs(sidewind_track) | 10 | 214.89 | 127.56 | 0.00 | 0.27 | 0.22 | 0.04 |
| ~ biome + abs(sidewind_track) | 7 | 234.88 | 147.54 | 0.00 | 0.18 | 0.16 | 0.02 |
| ~ tailwind_track * travel_hrs | 9 | 269.07 | 181.73 | 0.00 | 0.38 | 0.36 | 0.01 |
| ~ abs(sidewind_track) + travel_hrs | 7 | 275.50 | 188.16 | 0.00 | 0.27 | 0.22 | 0.04 |
| ~ season_spring + biome + travel_hrs | 7 | 282.05 | 194.72 | 0.00 | 0.37 | 0.36 | 0.01 |
| ~ season_spring + abs(sidewind_track) | 6 | 283.86 | 196.53 | 0.00 | 0.08 | 0.07 | 0.01 |
| ~ season_spring * tailwind_track | 9 | 288.80 | 201.47 | 0.00 | 0.19 | 0.18 | 0.01 |
| ~ (tailwind_track + abs(sidewind_track)) * season_spring | 11 | 366.34 | 279.01 | 0.00 | 0.20 | 0.19 | 0.01 |
| ~ biome + tailwind_track + abs(sidewind_track) | 8 | 431.56 | 344.23 | 0.00 | 0.27 | 0.27 | 0.01 |
| ~ tailwind_track + abs(sidewind_track) | 7 | 464.16 | 376.83 | 0.00 | 0.18 | 0.17 | 0.01 |
| ~ season_spring + tailwind_track | 6 | 473.35 | 386.02 | 0.00 | 0.18 | 0.17 | 0.01 |
| ~ season_spring + tailwind_track + biome | 7 | 474.14 | 386.80 | 0.00 | 0.28 | 0.28 | 0.01 |
| ~ (tailwind_track + abs(sidewind_track)) * travel_hrs * season_spring | 24 | 498.00 | 410.67 | 0.00 | 0.40 | 0.39 | 0.01 |
| ~ (tailwind_track + abs(sidewind_track)) * biome * season_spring | 16 | 513.76 | 426.43 | 0.00 | 0.30 | 0.29 | 0.01 |
| ~ season_spring * travel_hrs | 9 | 525.19 | 437.86 | 0.00 | 0.30 | 0.29 | 0.01 |
| ~ (tailwind_track + abs(sidewind_track)) * travel_hrs + season_spring | 15 | 546.05 | 458.72 | 0.00 | 0.40 | 0.39 | 0.01 |
| ~ (tailwind_track + abs(sidewind_track)) * biome | 11 | 547.86 | 460.53 | 0.00 | 0.28 | 0.27 | 0.01 |
| ~ biome * tailwind_track | 9 | 549.16 | 461.83 | 0.00 | 0.28 | 0.27 | 0.01 |
| ~ travel_hrs + tailwind_track + abs(sidewind_track) | 8 | 551.22 | 463.89 | 0.00 | 0.38 | 0.36 | 0.02 |
| ~ season_spring + abs(sidewind_track) + travel_hrs | 7 | 618.05 | 530.72 | 0.00 | 0.31 | 0.30 | 0.01 |
| ~ biome + travel_hrs | 6 | 623.89 | 536.56 | 0.00 | 0.33 | 0.31 | 0.03 |
| ~ abs(sidewind_track) | 5 | 624.46 | 537.13 | 0.00 | 0.04 | 0.00 | 0.04 |
| ~ tailwind_track * abs(sidewind_track) | 10 | 677.58 | 590.25 | 0.00 | 0.18 | 0.17 | 0.01 |
| ~ season_spring * abs(sidewind_track) | 9 | 699.48 | 612.14 | 0.00 | 0.08 | 0.07 | 0.01 |
| ~ biome + tailwind_track + travel_hrs | 8 | 701.70 | 614.37 | 0.00 | 0.42 | 0.41 | 0.01 |
| ~ biome * abs(sidewind_track) | 9 | 739.17 | 651.84 | 0.00 | 0.18 | 0.15 | 0.02 |
| ~ tailwind_track + travel_hrs | 7 | 744.57 | 657.24 | 0.00 | 0.38 | 0.36 | 0.01 |
| ~ season_spring + tailwind_track + travel_hrs | 7 | 756.60 | 669.26 | 0.00 | 0.39 | 0.38 | 0.01 |
| ~ travel_hrs | 6 | 762.76 | 675.43 | 0.00 | 0.26 | 0.22 | 0.04 |
| ~ season_spring + travel_hrs | 6 | 770.02 | 682.68 | 0.00 | 0.30 | 0.29 | 0.01 |
| ~ season_spring + biome + abs(sidewind_track) | 7 | 771.79 | 684.46 | 0.00 | 0.21 | 0.21 | 0.01 |
| ~ biome | 5 | 782.82 | 695.49 | 0.00 | 0.18 | 0.16 | 0.02 |
| ~ biome + tailwind_track | 7 | 801.62 | 714.29 | 0.00 | 0.27 | 0.27 | 0.01 |
| ~ season_spring * biome | 8 | 803.33 | 715.99 | 0.00 | 0.22 | 0.22 | 0.01 |
| ~ (tailwind_track + abs(sidewind_track)) * travel_hrs | 11 | 808.50 | 721.17 | 0.00 | 0.38 | 0.37 | 0.02 |
| ~ season_spring + tailwind_track + abs(sidewind_track) | 7 | 987.56 | 900.23 | 0.00 | 0.19 | 0.18 | 0.01 |
| ~ season_spring + biome | 6 | 989.40 | 902.07 | 0.00 | 0.21 | 0.20 | 0.01 |
| ~ season_spring | 5 | 1001.48 | 914.15 | 0.00 | 0.07 | 0.06 | 0.01 |
| ~ tailwind_track | 5 | 1097.88 | 1010.55 | 0.00 | 0.18 | 0.16 | 0.01 |
|  | 4 | 1101.02 | 1013.69 | 0.00 | 0.04 | 0.00 | 0.04 |

We tested an exhaustive set of models including fixed effects of tailwind and sidewind along the realized travel direction, daily travel time, season, their additive effects, and interaction effects between wind variables and daily travel time, biome and season. We further allowed intercepts to vary randomly between individuals and between years. Models are ranked according to increasing ∆AIC/decreasing AIC weight values, with the best performing model on top.
