# Supplementary material for: Adaptive drift and barrier-avoidance by a fly-forage migrant along a climate-driven flyway
Source: Mov Ecol. 2021 Jul 13;9:37. doi: 10.1186/s40462-021-00272-8 (PMC8276455; doi:10.1186/s40462-021-00272-8)
Supplement: Supplementary file 3 — Additional file 3. Codes for reproducing seasonal NDVI maps in Google Earth Engine. [file 40462_2021_272_MOESM3_ESM.docx]

*Vansteelant et al. (2021) Movement Ecology*

**Appendix 1:** Google Earth Engine code for producing Maximum Pixel Value Composites for NDVI for autumn and spring migration periods of Canarian Eleonora’s falcons

**CODE FOR AUTUMN COMPOSITE:**

// Load MODIS 16day NDVI composites for autumn: date ranges are mean start and end date of migration

var collection = ee.ImageCollection('MODIS/MCD43A4_006_NDVI')

.filter(ee.Filter.calendarRange(2012,2019,'year'))

.filter(ee.Filter.calendarRange(286,321,'day_of_year'))

.filter(ee.Filter.bounds(domain));

// Check metadata

print('All metadata:', collection);

// Check projection of NDVI data

var ndviProjection = ee.Image(collection.first()).select('NDVI').projection();

print('NDVI projection:', ndviProjection);

// Get scale (in meters) information from NDVI data

var ndviScale = ee.Image(collection.first()).select('NDVI').projection().nominalScale();

print('NDVI scale:', ndviScale); // ee.Number

// Create a greenest pixel composite.

var greenestPixelComposite = collection.qualityMosaic('NDVI');

// scale and projection.

var meanGreenest = greenestPixelComposite

// Request the data at the scale and projection of the MODIS image.

.reproject({

crs: ndviProjection

})

// Force the next reprojection to aggregate instead of resampling.

.reduceResolution({

reducer: ee.Reducer.max(),

maxPixels: 1000

});

// Display the results.

var vizParams = {bands: ['NDVI'], min: 0, max: 1, palette: ['black','red', 'orange', 'yellow', 'green']};

var map = meanGreenest.clip(domain).visualize(vizParams);

// Export image for further use in R

Export.image.toDrive({

image: meanGreenest,

description: 'Greenest_pixel_composite_autumn-fullb',

scale: 1000,

region: domain

});

**CODE FOR SPRING COMPOSITE:**

// Load MODIS 16day NDVI composites for spring: date ranges are mean start and end date of migration

var collection = ee.ImageCollection('MODIS/MCD43A4_006_NDVI')

.filter(ee.Filter.calendarRange(2013,2020,'year'))

.filter(ee.Filter.calendarRange(99,135,'day_of_year'))

.filter(ee.Filter.bounds(domain));

// Check metadata

print('All metadata:', collection);

// Check projection of NDVI data

var ndviProjection = ee.Image(collection.first()).select('NDVI').projection();

print('NDVI projection:', ndviProjection);

// Get scale (in meters) information from NDVI data

var ndviScale = ee.Image(collection.first()).select('NDVI').projection().nominalScale();

print('NDVI scale:', ndviScale); // ee.Number

// Create a greenest pixel composite.

var greenestPixelComposite = collection.qualityMosaic('NDVI');

// scale and projection.

var meanGreenest = greenestPixelComposite

// Request the data at the scale and projection of the MODIS image.

.reproject({

crs: ndviProjection

})

// Force the next reprojection to aggregate instead of resampling.

.reduceResolution({

reducer: ee.Reducer.max(),

maxPixels: 1000

});

// Display the results.

var vizParams = {bands: ['NDVI'], min: 0, max: 1, palette: ['black','red', 'orange', 'yellow', 'green']};

var map = meanGreenest.clip(domain).visualize(vizParams);

// Export image for further use in R

Export.image.toDrive({

image: meanGreenest,

description: 'Greenest_pixel_composite_spring-fullb',

scale: 1000,

region: domain

});
